# The effects of delayed auditory feedback depend on both the rhythm and content of the feedback signal

**DOI:** 10.3389/fnhum.2026.1889468

**Published:** 2026-09-16

**Authors:** Jason W. Bohland, Alexander Ocampo, Caitlin A. Lassige, Tess N. Freed, Matthias Heyne

**Affiliations:** 1 Department of Communication Science and Disorders, University of Pittsburgh, Pittsburgh, PA, United States; 2 Department of Bioengineering, University of Pittsburgh, Pittsburgh, PA, United States; 3 Center for the Neural Basis of Cognition, Carnegie Mellon University and the University of Pittsburgh, Pittsburgh, PA, United States; 4 Department of Communication Disorders, State University of New York at New Paltz, New Paltz, NY, United States

**Keywords:** delayed auditory feedback, feedback control, sensorimotor integration, speech, speech errors, speech production, speech rhythms, speech sequencing

## Abstract

**Purpose:**

Delayed auditory feedback (DAF) has long been known to disrupt fluent speech, yet the underlying mechanisms remain a matter of debate. Some have hypothesized that interference occurs because of asynchronous sensory and motor rhythms, independent of the specific content of auditory inputs. The goal of this study was to compare the effects of canonical DAF with those of delayed envelope modulated noise (EMN), a manipulation that preserves the speech envelope but removes other speech content. We hypothesized that canonical DAF would disrupt speech more than delayed EMN, revealing an important role for speech-related spectral content in feedback-driven interference of speech.

**Methods:**

38 neurologically healthy adults with no history of speech, language, or hearing disorders produced two types of speech materials (non-lexical 3-syllable sequences and sentences) under five auditory feedback conditions (speech feedback with minimal or 200 ms delay, EMN with minimal or 200 ms delay, and constant speech-shaped noise). Primary outcome measures were speech rate and error probability, with secondary measures including fundamental frequency, intensity, and within-trial modulation of each. Audio recordings were transcribed and analyzed using mixed-effects models for each outcome measure and stimulus type.

**Results:**

For each outcome measure and stimulus type, the effect of delay differed significantly between the SPEECH and EMN conditions. Speech feedback with 200 ms delay elicited large reductions in speech rate (~0.8–0.9 syllables/s) and large increases in error probability (~31–44%), which were consistent across stimulus types. EMN with 200 ms delay, on the other hand, elicited much smaller effects, with slight increases in speech rate (~0.13–0.14 syllables/s) and no significant changes in error rate. Delayed speech also caused increases in intensity and intensity modulation and decreases in pitch modulation, whereas results were less consistent for delayed EMN.

**Conclusion:**

These findings suggest that the presence of speech-related spectral content in the auditory feedback signal, above and beyond the asynchrony of speech rhythms, is necessary to elicit the full disruptive effects of DAF. We describe study results within a theoretical framework that includes roles for both rhythmic asynchrony and feedback content in explaining these disruptions.

## 1 Introduction

Under most circumstances, producing speech involves the continuous, sequential activation of the auditory system as the speaker hears the acoustic signals that result from their motor actions. The coupled sensory-motor system is impressive in the ease with which it facilitates rapid, fluent speech, while detecting and enabling the correction of errors even in noisy environments or situations with multiple competing talkers. One experimental manipulation that often severely disrupts the normal operation of this coupled system is delayed auditory feedback (DAF), in which the self-generated auditory feedback signal is artificially delayed (typically by 100–200 ms) before being transmitted to the speaker via headphones (Lee, 1950; Black, 1951). Under such conditions, both the segmental and suprasegmental aspects of speech are impacted. Commonly observed effects include induced disfluencies, individual sound or syllable repetitions or other speech errors, changes in intensity and pitch, and reductions in speech rate and pitch variation (Yates, 1963; Corey and Cuddapah, 2008; Chon et al., 2013; Malloy et al., 2022). While this simple experimental manipulation has been studied for decades, the specific underlying mechanisms by which it causes these changes remain relatively poorly understood.

One enduring hypothesis is that the disruption to speech caused by DAF is not due to active monitoring of the incoming speech sounds for content or the speaker’s attempts to use these signals for feedback-based control, but instead due to the *interference* between the speaker’s rhythmic speech output and a second rhythmic but temporally delayed auditory input signal. The Displaced Rhythm Hypothesis (DRH; Howell et al., 1983) formalized this idea, suggesting that only “the rhythmic properties of the syllable units, not their identity” drive these disruptions. The DRH shares similarities with other proposals that suggest that perception and action are coordinated through relative timing of motor actions and their resulting percepts (Pfordresher and Benitez, 2007).

The DRH has been partly supported by experimental studies that show reductions in speaking rate similar to those seen with DAF under conditions that modify the feedback signal. Howell et al. (1983) used gated speech feedback, in which a continuous square wave with fundamental frequency varying from 1 to 10 Hz turned auditory feedback (veridical speech without delay) on and off repeatedly while participants spoke random lists of vowels (/i/ and /u/). They found a significant effect on performance time (slowing of speech) for gated speech, with maximal disruption using a 2 Hz gating signal and attributed this disruption to changes in the *rhythm* of the feedback signals. In the same study, 5 participants were tested on a condition termed *part-delay, amplitude-restored*, in which a filtered version of the speech signal was gated on and off by a delayed estimate of the speech envelope (delays ranging from 0 to 400 ms). The results showed a significant effect of delay on reading time, with the maximum reading time at 200 ms delay, similar to typical DAF, leading the authors to suggest that the “rhythmic relationship of the delayed signal to the speech causes disruption because, even if the phonetic identity of the speech is destroyed, as much disturbance is observed as with standard DAF” (Howell et al., 1983, p. 782).

Howell and Archer (1984) further tested the DRH using a non-speech auditory feedback signal. Ten female participants produced random lists of vowels (/i/ and /u/) while exposed to speech or nonspeech auditory feedback. In the nonspeech condition, the speech envelope was isolated from the microphone signal, fed through a linear multiplier, and multiplied by a 500 Hz square wave (effectively replacing the phonetic detail with an envelope-modulated complex tone). Delays of 0–400 ms were introduced. This study found that the disruption (again measured via performance time) was equivalent to that caused by standard DAF, with maximal disruption at 200 ms delay.

Kaspar and Rübeling (2011) tested the influence of displaced speech rhythms using a paradigm in which participants repeated pairs of consonant-vowel (CV) syllables that varied in phonemic content and/or rhythm, with feedback delays varying in 0, 200, or 400 ms, with a dependent variable of reading time. They found all sequences to be significantly slowed with 200 ms delay, but reading times increased more for the rhythmic pairs (stress placed on one syllable) than the non-rhythmic pairs, which was consistent with predictions of the DRH. Further, they did not find a difference in reading time for the sequences varying in phonemic content vs. repeating sequences. It is notable, however, that all feedback contained veridical speech sounds (cf. Howell et al., 1983; Howell and Archer, 1984). These studies together provide converging support for the DRH, suggesting that the rhythmic properties of the auditory feedback signal are important components of speech disruption. The extent to which the phonetic or other speech-specific content of the feedback signal is also important (relative to just the rhythm) remains understudied, with direct comparisons of speech and non-speech feedback signals within the same participants lacking.

The present study is part of a larger investigation of the effects of DAF (Heyne et al., 2025), in which we sought to more specifically and directly test the DRH. Similar to Howell and Archer (1984) but using a software-based approach, we developed a method to provide auditory feedback signals that followed the produced speech envelope, but which lacked specific speech-related spectral content. Specifically, we used delayed envelope modulated noise (EMN) in place of the auditory feedback signal and compared the effects directly with those of canonical DAF (in which the speech signal is intact) in the same participants. We measured the effects of these feedback alterations with two primary articulatory/segmental outcome measures: speech rate and error rate. These measures are based on a large previous literature demonstrating rate reductions and increases in speech errors in typical adults subjected to DAF (Yates, 1963; Corey and Cuddapah, 2008; Chon et al., 2013; Malloy et al., 2022). We further measured changes in fundamental frequency and intensity, and within-trial variation in each. These suprasegmental measures are based on less consistent findings^1^ of increased vocal pitch (Fairbanks, 1955; Lechner, 1979; Ozker and Hagoort, 2025) and flattening of prosodic variation (Fairbanks, 1955; Chesters et al., 2015).

Based on previous work leading to a theoretical model of speech errors under DAF (Malloy et al., 2022), we hypothesized that canonical DAF would cause more disruptive effects (larger changes in the dependent variables) compared to delayed EMN. Our hypothesis is based on the notion that the displaced rhythm associated with DAF (phase relationship between motor signals and auditory inputs) is responsible for feedback arriving during time windows of unexpected excitability within the auditory cortex, but that the *content* of the auditory input remains critical for triggering specific speech errors and increased interference during speech motor planning and execution. The results reported here constitute a test of the DRH, specifically focusing on the importance of the displaced rhythmic envelope, and provide strong counterevidence to the claim that asynchrony between the speech output signal and arriving auditory feedback signal is the sole driver of DAF-induced speech disruptions.

## 2 Materials and methods

### 2.1 Participants

39 adult participants completed the set of experiments described here. These participants are a subset of a larger group who completed additional related tasks as described in Heyne et al. (2025). One participant’s data were excluded from the analysis due to a prodigious number of speech errors across all conditions, indicating an inability to successfully complete the task requirements. The remaining 38 participants (10 male, ages 18–60, *μ* = 30.8 yrs., σ = 10.9 yrs) provided the data used here. All participants self-reported as native American English speakers, with healthy, normal voice, speech, and hearing and passed a pure tone hearing screening (at 25 dB HL for octave frequencies between 250 Hz and 8 kHz separately in each ear), which was administered in a double-wall sound-treated booth using a calibrated Otometrics Madsen Astera audiometer and foam insert earphones. Exclusion criteria included previous treatment for a speech, language, or hearing disorder, or a history of neurological disease or stroke.

The study was approved by the University of Pittsburgh Institutional Review Board. Written informed consent was obtained from all participants prior to any study procedures.

### 2.2 Equipment

All testing was performed inside a sound-treated room. Participants viewed visual stimuli on a 24-inch monitor (VIEWPixx/EEG, VPixx Technologies) placed at a distance of ~1.5 m. Participants’ speech was recorded using a flexible, omnidirectional head-worn condenser microphone (Shure MX153; Shure, Inc.) positioned at ~7 cm distance, approximately 45° off axis from the center of the mouth. Auditory signals were delivered to participants via closed-back, dynamic, wired circumaural headphones, which provided up to 32 dB of passive noise reduction (Sennheiser HD 280 Pro, Sennheiser Group). Signals were processed via an RME Fireface UFX + audio device (RME Audio) with sampling rate set to 48 kHz, connected to a Windows workstation via USB 3.0. An additional amplifier (Rolls RA163; Rolls Corporation) was used at the output stage to deliver an amplified signal to the headphones (approximately 5 dB above the microphone input level). The microphone and simultaneous headphone signals were transmitted via digital optical cable to a multi-channel PCI audio card (RME HDSP 9632; RME Audio) onboard a separate Windows-based workstation. Signals were digitally recorded at 48 kHz sampling rate using Audacity software^2^. Figure 1 illustrates the basic experimental setup.

**Figure 1:**
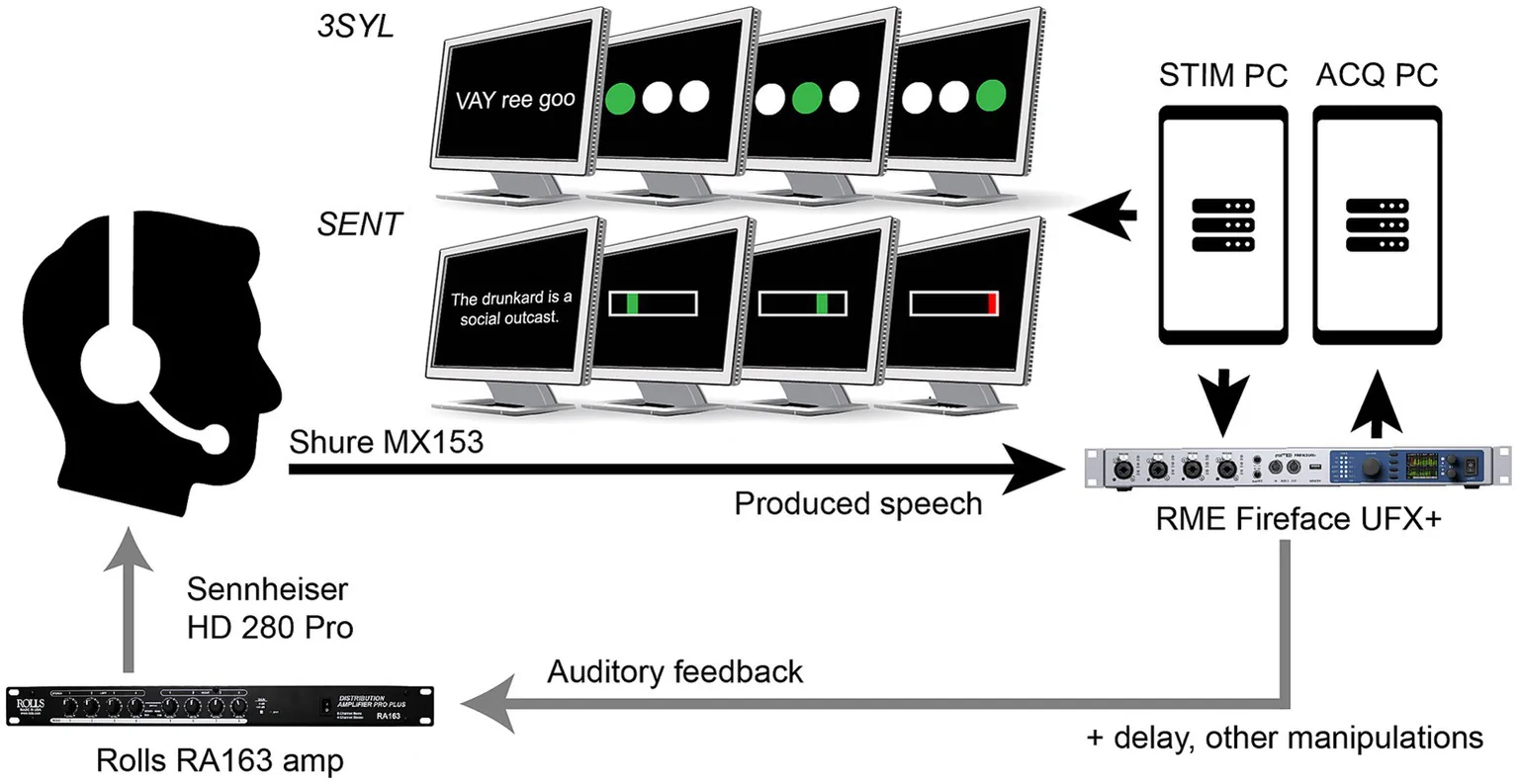
Illustration of the experimental setup used in this study. Top center shows visual displays for 3SYL and SENT tasks. The RME Fireface UFX + served as the main sound device, attached to the stimulus delivery computer (STIM PC), receiving microphone input from the participant. Custom code manipulated auditory feedback, which was sent to the participant headphones via the Rolls RA163 amplifier. Recording of the microphone and concurrent feedback signals was performed via optical cable on a separate workstation (ACQ PC). Image source: https://rme-audio.de; https://rolls.com/.

### 2.3 Stimuli

During each experimental session, participants completed several tasks, not all of which are described here (see Heyne et al., 2025). The tasks of interest for this investigation involved speaking prescribed utterances with different types of auditory feedback. The speech stimuli consisted of (1) three-syllable sequences (*3SYL*) consisting of CV, CVC, and VC (C = consonant, V = vowel) syllable frames, and (2) sentences (*SENT*) chosen from the Defense Advanced Research Projects Agency (DARPA) Texas Instruments – Massachusetts Institute of Technology (TIMIT; Garofolo et al., 1993) speech corpus.

#### 2.3.1 3SYL stimuli

Syllable sequences were composed from the following sounds: vowels (/a/, /i/, /u/, /eɪ/); consonants (/b/, /d/, /g/, /m/, /v/; /r/ in onset position only). Stimuli for each trial were selected pseudo-randomly from the full set of combinations, sampling consonants and vowels in equal numbers without repetition of sounds in adjacent syllables. On each trial, one sequence was presented orthographically (e.g., “VAY ree goo”) on the monitor for 2 s, with primary stress assigned to one pseudorandomly chosen syllable as indicated by upper case letters.

Participants were asked to repeatedly produce the syllable sequence, guided by a “visual metronome” (a series of circles that sequentially changed colors; see Malloy et al., 2022). This signal was intended to encourage speakers to attempt to maintain a steady, consistent, rate and rhythm, with a 200 ms inter-syllable onset interval. The recording period was 3,200 ms for each *3SYL* trial.

#### 2.3.2 SENT stimuli

100 sentences and corresponding audio recordings were selected from the TIMIT corpus. The chosen sentences were subject to several constraints: 4–7 words in length; spoken duration below the 80th percentile across all of TIMIT; dialects included Northern, North Midland, South Midland, New York City, Western, and “Army Brat” (indicating the speaker moved around the United States). The specific sentence sets were chosen using a greedy algorithm that minimized the correlation between three parameters in the set: (i) average number of syllables per word (a proxy for word complexity), (ii) the number of consonant clusters per syllable (a proxy for articulatory complexity), (iii) and the number of syllables per second in the selected audio exemplar (speech rate). Sentences were presented orthographically and, simultaneously, aurally, providing a template for the rate and rhythm the participant should target during production. During the production period, a “progress bar” moved from left to right, with the movement duration matched to the auditory exemplar. The recording period for each *SENT* trial was equal to the length of the auditory exemplar plus one second.

#### 2.3.3 Auditory feedback conditions

Participants were recorded while speaking the stimuli described above under different auditory feedback conditions as summarized in Table 1. Auditory feedback manipulations were controlled through custom MATLAB code using PsychPortAudio, a MATLAB-based wrapper for the PortAudio API, built for Psychophysics Toolbox Version 3 (PTB-3; Kleiner et al., 2007). This allowed us to achieve precise and accurate delays and low-latency signal processing using the Audio Stream Input/Output (ASIO) driver protocol coupled with the RME audio device.

**Table 1:** Auditory feedback conditions analyzed in this study.

Feedback condition	Description
SPEECH0	Unaltered speech (microphone) signals presented with minimal (~12 ms) delay
SPEECH200	Unaltered speech (microphone) signals presented with a specified 200 ms delay
EMN25	Speech-shaped noise modulated by the speech envelope and presented with a specified 25 ms delay
EMN200	Speech-shaped noise modulated by the speech envelope and presented with a specified 200 ms delay
NOISE	A continuous sample of speech-shaped noise was presented

The SPEECH0 and SPEECH200 conditions were extracted from blocks that also included trials with delays set to 150 or 250 ms. These delays were not included in the EMN blocks and thus are not included for analysis in this study.

For the SPEECH0 condition, which served as the most typical speech feedback condition, a special *Direct Input Monitoring* mode was used to achieve minimal delays, which were measured to be ~12 ms between the microphone and headphone signals, using the method described by Kim et al. (2020). Figure 2 shows feedback signals used across SPEECH, EMN, and NOISE conditions.

**Figure 2:**
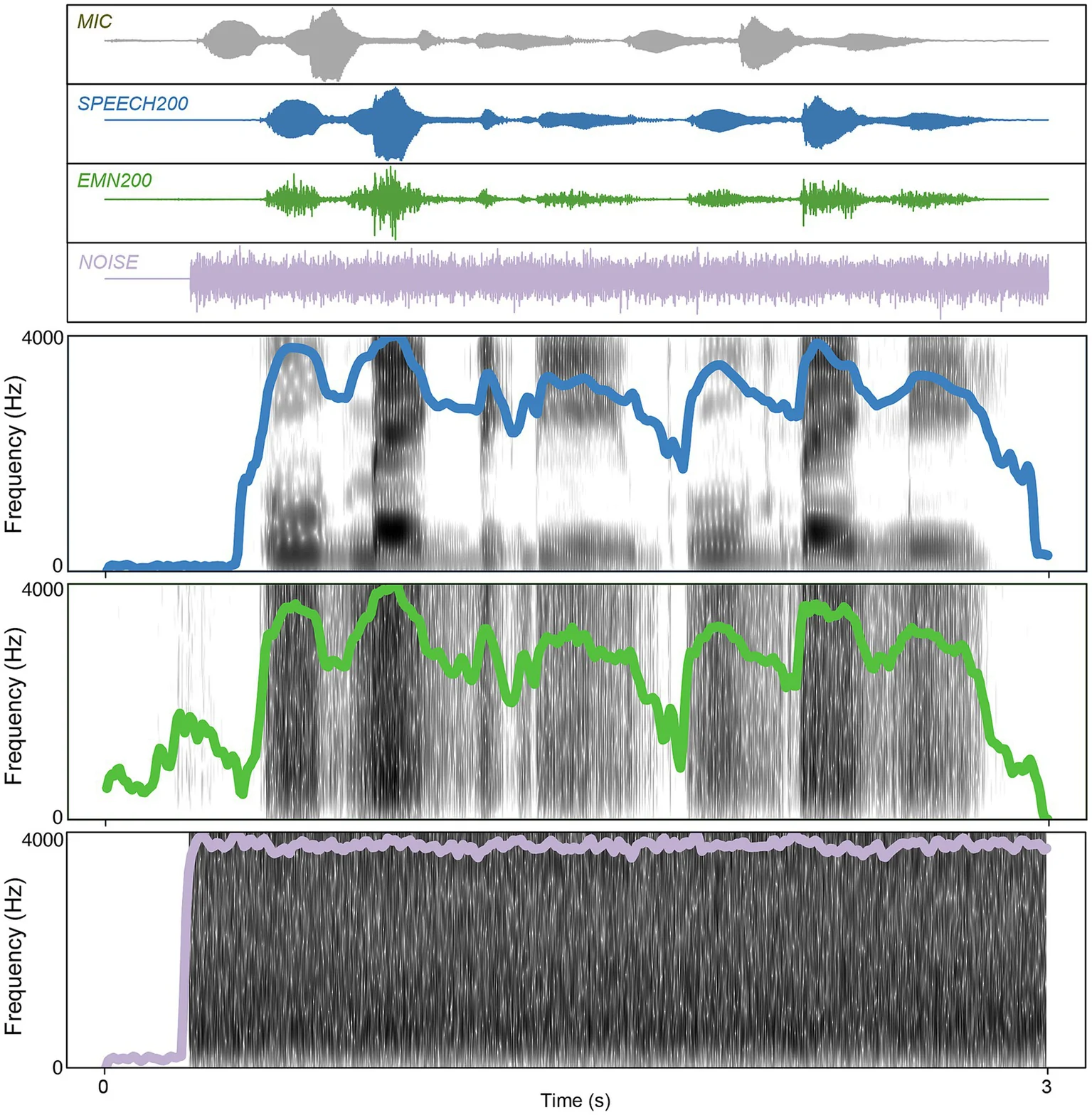
Illustration of auditory feedback conditions. Top: Time-domain waveforms from an example *3SYL* trial. Shown are the microphone signal (gray), the SPEECH200 feedback signal (blue; preserved waveform delayed by 200 ms), the EMN200 feedback signal (green; preserved envelope used to modulate speech-shaped noise, delayed by 200 ms), and the NOISE signal (purple; constant speech-shaped noise). Bottom: Spectrograms for the SPEECH200, EMN200, and NOISE feedback signals with overlaid intensity contours estimated in Praat. Contours illustrate very similar rhythm and amplitude contours between SPEECH200 and EMN200 conditions, but the signals have different spectral profiles, eliminating all phonetic content in the EMN200 feedback signal.

#### 2.3.4 Speech shaped noise

The long-term power spectrum of speech was estimated by applying the Fast Fourier Transform (FFT) to a concatenated set of 150 sentences selected from the TIMIT database^3^. The phase of each frequency component of the resulting FFT was then randomized, which eliminates the original temporal structure while preserving the same amplitude spectrum. The inverse FFT was used to obtain a long duration (~320 s) sample of speech-shaped noise (SSN).

For the conditions using speech shaped noise (NOISE, EMN25, and EMN200; see Table 1), the RME audio device buffer was set to 64 samples. For the EMN25 and EMN200 conditions, we programmatically accessed the input (microphone buffer), processed it using custom code, and wrote the results to the output buffer with a delay of either 25 ms^4^ (EMN25) or 200 ms (EMN200). Custom MATLAB code was developed to obtain the speech envelope over short windows and multiply it by a segment of the SSN sample. Specifically, the root mean square (RMS) envelope of the microphone signal was extracted using a 256 sample (5.33 ms) moving window. The resulting envelope was multiplied by an SSN sample of the same length. The noise samples used within a trial formed a continuous segment of the SSN sample, preserving the underlying autocorrelation structure. The same algorithm was used for both the *3SYL* and *SENT* stimuli. SSN-derived feedback signals were rescaled, based on a calibration procedure, to approximately match the +5 dB microphone-to-headphones closed loop gain used in SPEECH0 and SPEECH 200 conditions.

The EMN25 and EMN200 conditions largely preserved the envelope of the microphone signal (see Figure 2) but removed all phonetic and other speech-specific spectral content. These feedback conditions varied the phase relationship between the microphone and headphones in a manner that approximately mirrored the SPEECH0 and SPEECH200 conditions. In the NOISE feedback condition, participants were presented with a continuous segment of the SSN signal throughout the entire feedback period. This condition generally masked auditory feedback but contained no rhythmic envelope modulation of the sound input. It served as a control comparison for other conditions.

### 2.4 Procedure

All data analyzed in this study were collected during a single experimental session for each participant. Within each session, tasks were presented in blocks of ~6–8 min duration. *3SYL* and *SENT* stimuli were presented in separate blocks. Speech-based (SPEECH0 and SPEECH200) and noise-based (EMN25, EMN200, and NOISE) feedback conditions were presented in separate blocks for each stimulus type. All stimuli and auditory feedback conditions were pseudorandomized across trials within each block. Some participants (*N* = 23) previously completed an earlier session on a previous day involving similar DAF manipulations but not including the EMN or NOISE conditions. These data are not included in this study.

Participants completed one run each of *3SYL* and *SENT* stimulus types using noise-based feedback. Each *3SYL* run contained 60 trials: 15 trials with NOISE, 15 trials with EMN25, and 30 trials with EMN200 feedback. Each *SENT* run contained 50 trials: 12 trials with NOISE, 13 trials with EMN25, and 25 trials with EMN200 feedback.

Each participant also completed two runs of the same *3SYL* task with minimally delayed (SPEECH0) and delayed speech feedback. In those runs, feedback was delayed by 150, 200, or 250 ms (in pseudorandom order) on different trials. To directly compare the effects of delayed envelope modulated noise to delayed speech feedback for the *3SYL* sequence productions, we only used the 200 ms delay trials (SPEECH200 condition) in this analysis, which correspond to a subset of trials from within those runs. Across two runs, 30 trials with SPEECH0 feedback and 30 trials with SPEECH200 feedback were analyzed.

Finally, each participant also completed two runs of the *SENT* stimulus type with minimally delayed and delayed auditory feedback, again with pseudorandomly presented latencies varying from 150–250 ms in different trials. Across two *SENT* runs, 24 trials with SPEECH0 feedback and 24 trials with SPEECH200 feedback were analyzed.

### 2.5 Audio processing and transcription

Speech audio recordings were split into stereo .wav files representing individual trials (one channel for the microphone recording and the other for simultaneous auditory feedback) and checked for technical errors or missed productions; such trials (*N* = 3 *3SYL* and *N* = 5 *SENT*) were excluded from analyses.

A total of 4,554 *3SYL* trials and 3,719 *SENT* trials were further analyzed. Each participant’s data from a single run of one task were divided into two batches (*N = 29–36* trials, with ~20% overlap) and assigned to 1 of 11 trained listeners^5^ who performed a custom, streamlined transcription task using a Python-based graphical user interface (GUI). Listeners who were assigned a batch of the SPEECH conditions were also assigned a batch of the EMN/NOISE conditions for the same participant and stimulus type (*3SYL*/*SENT*); this facilitated within-participant consistency. Listeners were asked to set temporal markers indicating the start and end of the production (used for speech rate calculations) as well as the position of the first (if any) error. Correct productions of target words/syllables were marked by button presses within the GUI. Distortions and disfluencies were marked with special characters and incorrectly produced syllables (*3SYL* stimuli) or words/nonwords (*SENT* stimuli) were marked using specific annotations. Listeners could replay the whole trial or a selected portion of the trial as many times as needed to complete the transcription. Because listeners could see the filenames of individual trials, they were not fully blinded to the experimental conditions (i.e., they could determine if a trial was from a canonical DAF run or a run involving EMN and NOISE conditions, but they could not see the delay or specific feedback conditions). Transcriptions were exported and categorized as containing at least one error or being error free. For trials with more than one listener (*N* = 1,074 *3SYL* trials, *N* = 847 *SENT* trials), the trial was considered an error trial only if *all* listeners transcribed an error. Requiring unanimous listener agreement was a conservative analytic choice, motivated by a desire to limit false positive error annotations.

### 2.6 Inter-rater reliability of error annotations

Inter-rater reliability for the binary decision of whether or not a trial contained an error was measured using Gwet’s AC1 (Gwet, 2008). This measure was preferred over Cohen’s *kappa* and its multi-rater generalization (Fleiss, 1971) as it is less affected by differences in rating prevalence and marginal probability (Wongpakaran et al., 2013). In order to not only quantify listener agreement but also to test if agreement differed across experimental conditions, this robustness to prevalence (e.g., how frequently an error occurs) was important, since we expected error rates would vary across our designed experimental conditions.

The *gwet.ac1.raw*() function from the *irrCAC* R package (Gwet, 2026) was used to measure AC1 and average percent agreement separately for the SPEECH, EMN, and NOISE conditions for each of the *3SYL* and *SENT* stimulus types. For *3SYL* stimuli, there were 546 SPEECH trials, 389 EMN trials, and 139 NOISE trials with multiple listeners. For *SENT* stimuli, there were 407 SPEECH trials, 335 EMN trials, and 105 NOISE trials with multiple listeners. To determine if there were significant differences in listener agreement across the primary conditions of interest (*SPEECH* and *EMN*), we used a bootstrap approach, sampling trials from each condition with replacement, and calculating the between-condition AC1 difference from pairs of samples. A total of 5,000 bootstrap pairs were used, and we tested whether or not the central 95th percentile range of this distribution (between the 2.5^th^ percentile and 97.5^th^ percentile values) contained zero (i.e., no difference in listener agreement between conditions). This represented a non-parametric two-tail test with an alpha level of 0.05.

### 2.7 Pitch and intensity extraction

Pitch and intensity estimates were extracted from trial audio using the *parselmouth* Python library (Jadoul et al., 2018) for Praat (Boersma et al., 2001). Pitch (fundamental frequency; *F_0_*) was estimated with raw autocorrelation using a floor of 75 Hz and ceiling of 600 Hz, with 10 ms time step. Intensity contours (in dB^6^) were estimated using a minimum pitch value of 75 Hz and 10 ms time step. These estimates were intersected with the boundaries of speech production per trial determined by listeners (see above), resulting in vectors of *F_0_* and intensity estimates across the utterance. Time points with invalid *F_0_* or intensity estimates were excluded from calculations. *F_0_* and intensity modulation (per trial) were estimated as the standard deviation of the estimated values in that trial.

### 2.8 Statistical approach

Linear mixed effects models were used to analyze the continuous dependent variables: speech rate, fundamental frequency, fundamental frequency variability, intensity, and intensity variability. Models were estimated using the *lme4* R package (Bates et al., 2015) and included fixed categorical effects of condition (SPEECH, EMN, NOISE; reference level SPEECH), delay (minimal, 200 ms; reference level minimal), and their interaction, as well as covariates for sex (binary factor; reference level female), age (continuous, mean-centered) and trial number (continuous, mean-centered). Default treatment (dummy) coding was used, comparing each level of categorical factors to their reference baselines. In this formulation, the intercept refers to a female participant of mean age at the mean trial, in the SPEECH0 condition. Models included random intercepts by participant and random by-participant slope for delay. This model structure was determined based on likelihood ratio tests comparing models of increasing complexity fit via maximum likelihood to speech rate data, while also ensuring model convergence across different outcome measures. The same model structure was applied for all dependent variables. Final parameter estimates used restricted maximum likelihood (REML) estimation. Statistical significance was determined using the *lmerTest* R package (Kuznetsova et al., 2017) using the Satterthwaite method for estimating degrees of freedom. All models were visually inspected for gross violations of assumptions using the *DHARMa* R package (Hartig, 2024).

Generalized linear mixed effects models (using the *lme4* package) were used to analyze the binary dependent variable (occurrence of one or more speech errors) per trial. Models again included fixed effects of condition (SPEECH, EMN, NOISE), delay (minimal, 200 ms), and their interaction, as well as covariates for sex (binary factor), age (continuous, mean-centered), and trial number (continuous, mean-centered), random intercepts by participant, and random by-participant slope for delay. The same reference levels and contrast coding strategies applied to linear mixed effects models were used for the generalized linear mixed models. The *glmer* function was used to estimate these models, with family set to “binomial”; the *BOBYQA* (Bound Optimization by Quadratic Approximation; Powell, 2009) algorithm was used for optimization.

For all models, the unbalanced design (specifically, the NOISE condition was not crossed with delay) resulted in the model matrix being rank deficient, such that one model coefficient was dropped relative to a fully crossed design. The dropped term in all models corresponded to the absent NOISE x 200 ms delay cell. To aid the reader, we have annotated the model results tables to indicate how each estimated coefficient should be interpreted. The primary hypothesis tested for each model concerned the *condition* (SPEECH, EMN) x *delay* (minimal, 200 ms) interaction, testing whether the effect of delay differed between SPEECH and EMN conditions. For each model, this interaction term was directly estimated as a single coefficient and was evaluated using an uncorrected *p-*value. The interaction term represents the difference between the delay effect in the EMN condition and the delay effect in the SPEECH condition (i.e., [EMN200 – EMN25] – [SPEECH200 – SPEECH0]).

Additional contrasts of interest were estimated using the *emmeans* R package (Lenth and Piaskowski, 2026) to compute linear combinations of model coefficients. Within each model, these planned contrasts were treated as a family and corrected for multiple comparisons using the Bonferroni-Holm method. For each model, we tested the following contrasts:

1. SPEECH200 vs. SPEECH0. We hypothesized that canonical delayed auditory feedback would have a significant effect on all outcome variables.
2. EMN200 vs. EMN25. We had no strong *a priori* hypotheses about the effect of delay in the EMN condition; however, this contrast supported interpretation of any observed condition x delay interaction.
3. EMN200 vs. SPEECH0. This contrast determined whether delayed envelope modulated noise feedback elicited effects that differed from the condition closest to typical speech.
4. SPEECH0 vs. NOISE. This contrast determined the effects of constant speech-shaped noise feedback on each outcome variable relative to typical speech.
5. EMN25 vs. NOISE. This contrast determined how rhythmically modulated speech-shaped noise compared to the effects of constant speech-shaped noise.
6. EMN200 vs. NOISE. This contrast determined how the effects of delayed, rhythmically modulated speech-shaped noise compared to the constant speech-shaped noise masking condition.

In contrasts 4–6, the NOISE condition served as a control that removed speech-specific content but included no rhythmic modulation. These contrasts support interpretation of the primary results. The set of six contrasts above are presented as a separate table for each model, with family-wise error correction applied to *p-*values. Where a contrast coincides with a model coefficient directly (contrast 1 and contrast 4, though with opposite sign), the estimate and test-statistic are identical, but the *p-*value differs due to the application of the Bonferroni-Holm adjustment for the planned contrasts. All model coefficient tables report unadjusted *p*-values for all model terms, while all contrast tables report adjusted *p*-values within each model. Given the number of models evaluated (6 dependent variables x 2 stimulus types) and the multiple contrasts within each, secondary suprasegmental findings should be interpreted with appropriate caution.

## 3 Results

*3SYL* and *SENT* data were analyzed separately since the obtained results are not directly comparable due to differences in both stimulus presentation approach and data processing methods. Results for both stimulus types are reported below for each segmental and suprasegmental dependent variable.

### 3.1 Speech rate

Speech rate was extracted from all processed trials (see above) under all five auditory feedback conditions. Figure 3 shows box plots of the mean speech rate by speaker across conditions for both *3SYL* (left panel) and *SENT* (right panel) stimulus types.

**Figure 3:**
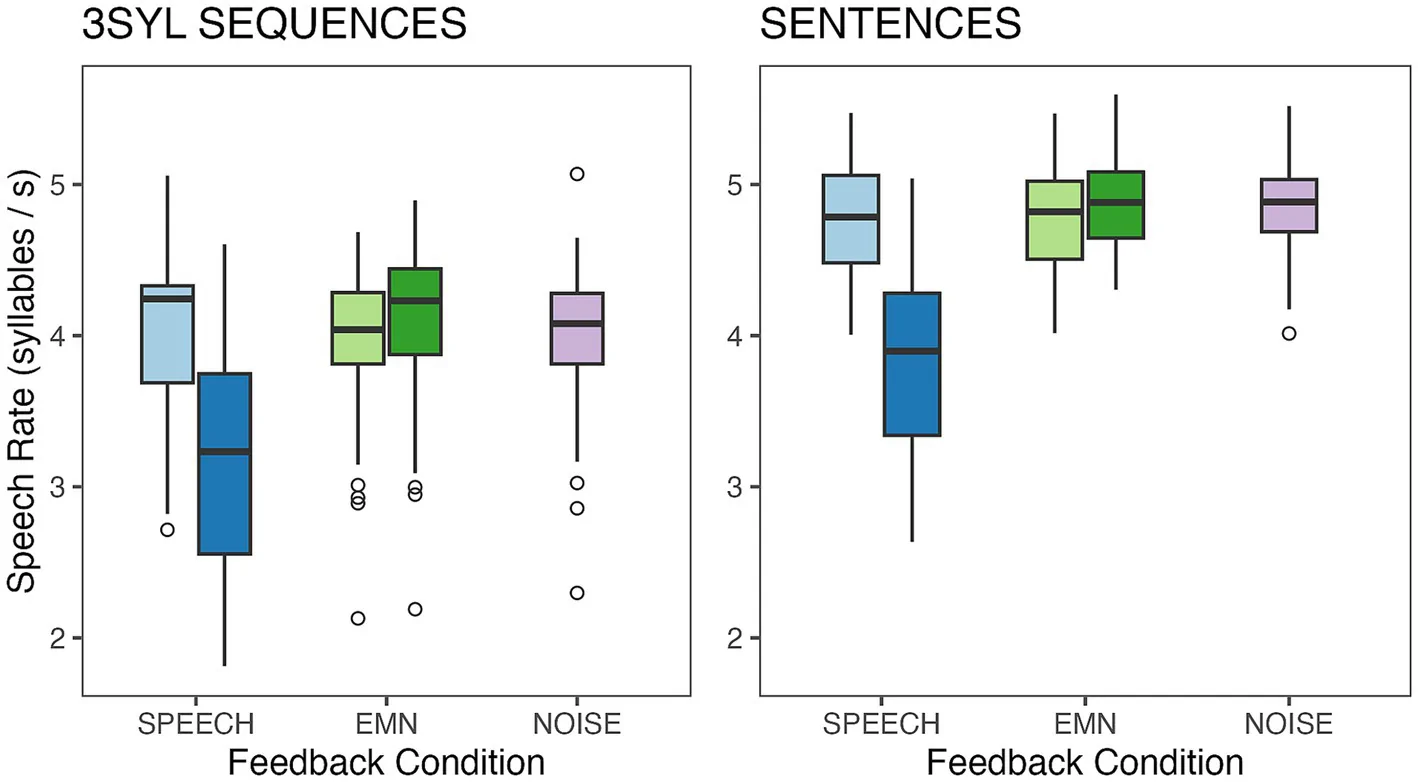
Estimated speech rate (syllables/second) across experimental manipulations. Left: Results from 3-syllable sequence stimuli. Right: Results from sentence stimuli. Darker shades (right boxes and whiskers in each pair) represent data from the 200 ms delay condition (SPEECH200, EMN200), while lighter shades (left boxes) represent data from the minimal delay conditions (SPEECH0, EMN25). Boxes span the interquartile (IQR) range; whiskers extend to 1.5 times the IQR, with circles indicating outlier participants. SPEECH conditions used speech auditory feedback; EMN conditions used envelope modulated noise auditory feedback. The NOISE condition used constant speech-shaped noise with no manipulation for delay.

Two linear mixed effects models were estimated to determine relationships with designed experimental manipulations (condition, delay, and their interaction) and covariates (age, sex, trial) and to assess statistical significance. In these and all statistical models, the SPEECH condition and minimal delay were treated as the reference conditions. Resulting model coefficients are shown in Table 2. The results of planned contrasts evaluated with *emmeans* are shown in Table 3.

**Table 2:** Linear mixed effects model estimates for speech rate.

Fixed effects	3SYL					SENT				
	Estimates	CI	*t*	df	*p*	Estimates	CI	*t*	df	*p*
Intercept [SPEECH0]	4.057	3.857–4.258	41.030	36.0	**<0.001**	4.733	4.605–4.861	74.533	40.1	**<0.001**
Condition [EMN25−SPEECH0]	−0.090	−0.138 – −0.041	−3.620	4474.0	**<0.001**	−0.007	−0.081 – 0.068	−0.181	3639.1	0.857
Condition [NOISE−SPEECH0]	−0.045	−0.094 – 0.003	−1.832	4474.0	0.067	0.102	0.026–0.178	2.619	3639.1	**0.009**
Delay [SPEECH200−SPEECH0]	−0.776	−0.871 – −0.682	−16.562	45.0	**<0.001**	−0.887	−0.980 – −0.793	−18.933	63.3	**<0.001**
Sex [MALE−FEMALE]	−0.204	−0.590 – 0.182	−1.074	35.0	0.290	0.123	−0.119 – 0.366	1.032	35.0	0.309
Age (centered)	−0.014	−0.030 – 0.001	−1.866	35.0	0.070	−0.010	−0.020 – −0.000	−2.101	35.0	**0.043**
Trial (centered)	−0.002	−0.003 – −0.002	−5.652	4484.6	**<0.001**	−0.001	−0.002 – 0.001	−0.765	3660.3	0.444
Condition x delay [EMN200−EMN25]−[SPEECH200−SPEECH0]	0.918	0.856–0.981	28.699	4474.0	**<0.001**	1.015	0.918–1.111	20.594	3639.1	**<0.001**

Random effects	*3SYL* variance	*SENT* variance
*σ2*	0.233	0.460
τ00 SUBJECT	0.269	0.096
τ11 SUBJECT.delay200	0.068	0.045
ρ01 SUBJECT	0.190	0.234
ICC	0.585	0.226
Marginal R2	0.209	0.235
Conditional R2	0.672	0.408

Separate models were fit for *3SYL* and for *SENT*. Reported *p-*values for individual fixed effects coefficients are not adjusted for multiple comparisons. Model formula: Rate ~ condition*delay + sex + age + trial + (1 + delay|subject). Bold values indicate an uncorrected *p* < 0.05.

**Table 3:** Results of planned contrasts for speech rate.

Contrast	3SYL					SENT				
	Estimate	SE	*t*	df	*p*_adj_	Estimate	SE	*t*	df	*p*_adj_
SPEECH200−SPEECH0	−0.776	0.047	−16.562	45.0	**<0.0001**	−0.887	0.047	−18.933	63.3	**<0.0001**
EMN200−EMN25	0.142	0.049	2.900	53.8	**0.027**	0.128	0.051	2.509	88.6	**0.047**
EMN200−SPEECH0	0.052	0.047	1.117	45.0	0.270	0.121	0.047	2.595	62.2	**0.047**
SPEECH0−NOISE	0.045	0.025	1.832	4474.0	0.204	−0.102	0.039	−2.619	3639.1	**0.044**
EMN25−NOISE	−0.044	0.029	−1.551	4474.0	0.242	−0.109	0.044	−2.470	3639.0	**0.047**
EMN200−NOISE	0.098	0.049	1.995	53.7	0.204	0.019	0.052	0.368	93.9	0.713

*P-*values are adjusted for multiple comparisons within family (the set of six contrasts, evaluated separately for *3SYL* and *SENT*) using the Bonferroni-Holm method. Bold values indicate an adjusted *p* < 0.05.

Consistent with our primary hypothesis, both the *3SYL* and *SENT* models revealed a significant interaction between condition and delay, such that the effect of increasing delay was different for SPEECH and EMN conditions (3SYL: *t*(4474.0) = 28.699*, p* < 0.001; SENT: *t*(3639.1) = 20.594, *p* < 0.001). Comparisons within condition using the *emmeans* R package showed statistically significant changes (*p* < 0.05; reported *p*-values adjusted for multiple comparisons within each model) in speech rate with delay for both SPEECH and EMN. For *3SYL* stimuli, participants significantly decreased their speech rate for SPEECH200 vs. SPEECH0 (estimated marginal mean difference = −0.776 syllables/s, *t*(45.0) = −16.562, *p_adj_ <* 0.0001) but *increased* their rate slightly for EMN200 vs. EMN25 (estimated marginal mean difference = 0.142, *t*(53.8) = 2.900, *p_adj_* = 0.027). For *SENT* stimuli, rate again decreased significantly for SPEECH200 vs. SPEECH0 (estimated marginal mean difference = −0.887 syllables/s, *t*(63.3) = −18.933, *p_adj_* < 0.0001) and *increased* for EMN200 vs. EMN25 (estimated marginal mean difference = 0.128, *t*(88.6) *= 2.509*, *p_adj_ = 0.047*). Comparing EMN200 vs. SPEECH0 revealed a small increase in speech rate (estimated marginal mean difference = 0.121 syl/s, *t*(62.2) = 2.595, *p_adj_ =* 0.047) for delayed envelope modulated noise relative to “natural” speech for *SENT* stimuli only.

For *SENT* stimuli, speech rate was significantly reduced for EMN25 vs. NOISE (estimated marginal mean difference = −0.109 syllables/s, *t*(3639.0) = −2.470, *p_adj_* = 0.047) and for SPEECH0 vs. NOISE (estimated marginal mean difference = −0.102 syllables/s, *t*(3639.1) = −2.619, *p_adj_* = 0.044), suggesting a slight tendency to speak more rapidly in the constant NOISE condition. No other contrasts with the NOISE condition were significant for either stimulus type.

### 3.2 Listener agreement

Each trial was categorized as being with or without at least one speech error (including sound substitutions, repetitions, omissions, severe distortions, or other disfluencies) based on listener transcriptions. While most trials were annotated by only one listener, a portion (*N* = 1,074 *3SYL* trials, *N* = 847 *SENT* trials) were annotated by two or more listeners. From these trials, Gwet’s AC1, a measure of inter-rater reliability (Gwet, 2008) was calculated, separately for each condition (SPEECH, EMN, and NOISE) and each stimulus type (*3SYL*, *SENT*). These values, which quantify agreement on the binary decision of whether a trial contains a speech error, are summarized in Table 4, which also includes the average pairwise percent agreement between listeners.

**Table 4:** Inter-rater reliability for the presence/absence of an error based on listener annotations.

Condition	3SYL			SENT		
	AC1	AC1 95% CI	% Agreement	AC1	AC1 95% CI	% Agreement
SPEECH	0.664	[0.601,0.727]	83.0%	0.749	[0.685,0.813]	86.2%
EMN	0.695	[0.623,0.767]	82.8%	0.813	[0.755,0.870]	86.9%
NOISE	0.805	[0.708,0.902]	88.5%	0.823	[0.721,0.924]	87.6%
SPEECH-EMN	−0.031	[−0.120, 0.064]	0.2%	−0.064	[−0.152, 0.023]	−0.7%

The 95% confidence interval for SPEECH-EMN was determined through a bootstrapping approach (see Methods).

Based on previously published interpretations of AC1 scores (Wongpakaran et al., 2013; Dettori and Norvell, 2020), listeners showed good or very good agreement in all conditions, with overall highest scores in the NOISE condition and lowest in the SPEECH condition. We used a bootstrap sampling approach to test if there was a statistically significant difference between AC1 scores for the SPEECH and EMN conditions, which were of primary interest. For *3SYL* stimuli, the 95% bootstrap confidence interval was [−0.120, 0.064], containing zero. The 95% bootstrap confidence interval for *SENT* stimuli was [−0.152, 0.023], also containing zero. Therefore, we concluded that, although inter-rater reliability was, on average, higher for EMN than for SPEECH, these differences were not statistically significant.

### 3.3 Error rate

Figure 4 shows box plots of the mean speech error rate by speaker across conditions for *3SYL* (left panel) and *SENT* (right panel) stimulus types.

**Figure 4:**
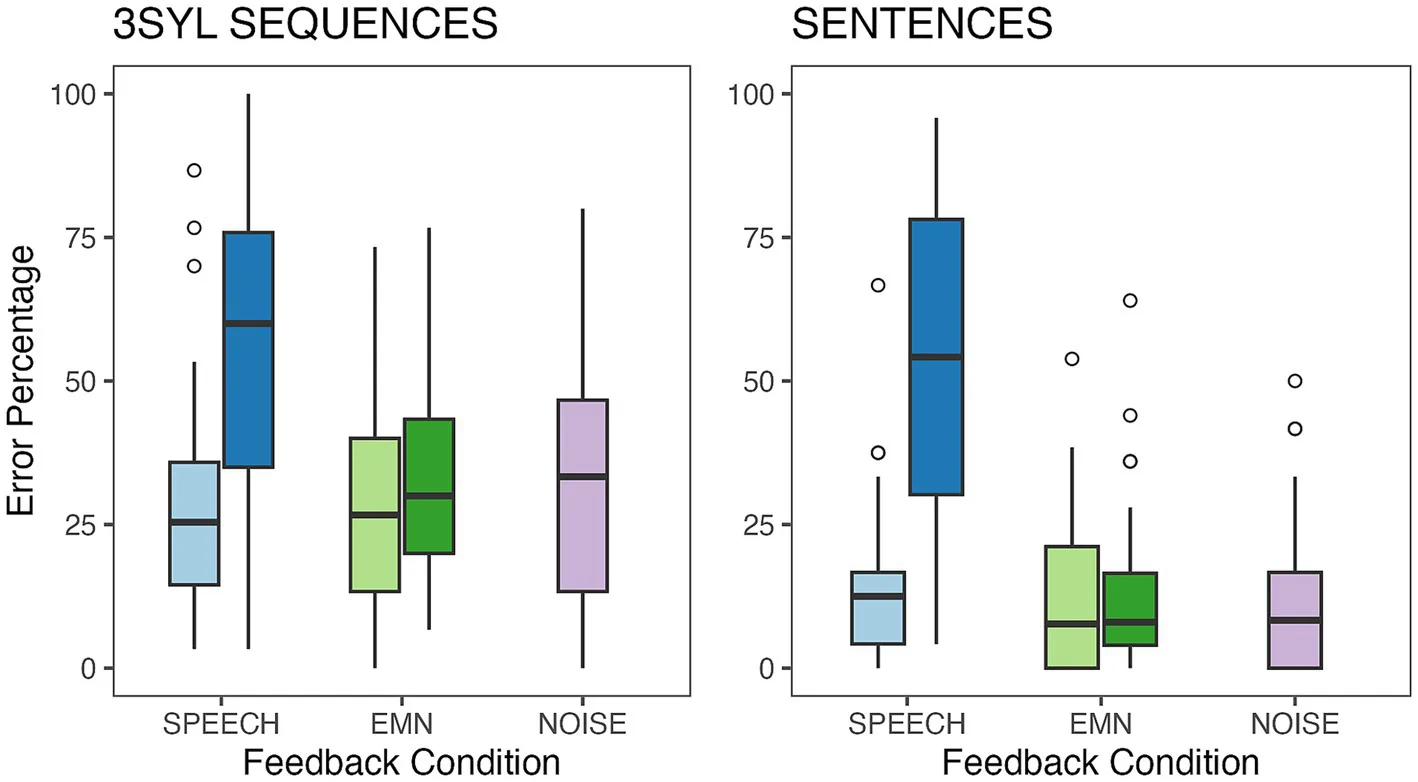
Error rate (percentage of trials with at least one speech error) across experimental manipulations. Left: Results from 3-syllable sequence stimuli. Right: Results from sentence stimuli. Darker shades (right boxes and whiskers in each pair) represent data from the 200 ms delay condition (SPEECH200, EMN200), while lighter shades (left boxes) represent data from the minimal delay conditions (SPEECH0, EMN25). Boxes span the interquartile (IQR) range; whiskers extend to 1.5 times the IQR, with circles indicating outlier participants.

Generalized linear mixed effects (logistic regression) models were fit separately for *3SYL* and *SENT* datasets. Table 5 shows model estimates, and Table 6 shows the results of planned contrasts, while transforming log-odds to more interpretable differences in predicted probabilities. Both models showed a significant interaction between delay and condition such that the effect of delay on error rate was different for SPEECH than for EMN (*3SYL*: z = −6.699, *p* < 0.001; *SENT*: *z* = −10.593, *p* < 0.001). As with speech rate, planned contrasts using *emmeans* showed a statistically significant change in error occurrence with delay in the SPEECH condition. For *3SYL*, the probability of error, after controlling for other predictors, during the SPEECH200 condition was estimated to be 31.07% higher than in the SPEECH0 condition (stderr = 2.31%, z-ratio = 13.454, *p_adj_* < 0.0001). The probability of error increased between EMN25 and EMN200, though the estimated magnitude of the change was much smaller and did not reach statistical significance when correcting for multiple comparisons (6.08%, stderr = 2.71%, *z*-ratio = 2.243, *p_adj_* = 0.100). For *3SYL*, there was a statistically significant increase in error rate for EMN200 vs. SPEECH0 (6.14%, stderr = 2.29%, *z*-ratio = 2.676, *p_adj_* = 0.037), suggesting that participants made more errors under delayed envelope modulated noise than typical, minimally delayed feedback. For *SENT* stimuli, the estimated increase in error rate between SPEECH0 and SPEECH200 was 44.05% (stderr = 4.01%, z-ratio = 10.989, *p_adj_* < 0.0001), while there was no significant change between EMN25 and EMN200 or between EMN200 and SPEECH0.

**Table 5:** Results from generalized linear mixed effects model estimates for error rate.

Fixed effects	3SYL				SENT			
	OR	CI	*z*	*p*	OR	CI	*z*	*p*
Intercept [SPEECH0]	0.298	0.214–0.416	−7.14	**<0.001**	0.098	0.067–0.143	−11.925	**<0.001**
Condition [EMN25 vs. SPEECH0]	1.003	0.792–1.271	0.025	0.980	0.897	0.640–1.256	−0.633	0.527
Condition [NOISE vs. SPEECH0]	1.218	0.966–1.536	1.664	0.096	0.89	0.630–1.257	−0.663	0.507
Delay [SPEECH200 vs. SPEECH0]	3.676	3.006–4.495	12.684	**<0.001**	8.597	6.318–11.699	13.687	**<0.001**
Sex [MALE vs. FEMALE]	1.936	1.077–3.481	2.208	**0.027**	2.65	1.392–5.042	2.968	**0.003**
Age (centered)	0.984	0.960–1.008	−1.336	0.182	1.007	0.979–1.036	0.518	0.605
Trial (centered)	1.000	0.996–1.003	−0.169	0.866	1.003	0.997–1.010	1.034	0.301
Condition x delay [EMN200 vs. EMN25] vs. [SPEECH200 vs. SPEECH0]	0.359	0.266–0.485	−6.699	**<0.001**	0.096	0.063–0.149	−10.593	**<0.001**

Random effects	*3SYL* variance	*SENT* variance
*σ2*	3.290	3.290
τ00 NSFDAF_ID	0.642	0.606
τ11 NSFDAF_ID.delay200	0.048	0.265
ρ01 NSFDAF_ID	−0.291	0.462
ICC	0.157	0.219
Marginal R^2^	0.091	0.218
Conditional R^2^	0.234	0.389

Separate models were fit for *3SYL* and for *SENT*. Reported *p-*values for individual fixed effects coefficients are obtained based on asymptotic Wald tests and are not adjusted for multiple comparisons. Model formula: Error ~ condition*delay + sex + age + trial + (1 + delay | subject). Bold values indicate an uncorrected *p* < 0.05.

**Table 6:** Results of planned contrasts for error rate.

Contrast	3SYL				SENT			
	Δ probability	SE	z	*p*_adj_	Δ probability	SE	z	*p*_adj_
SPEECH200−SPEECH0	0.311	0.023	13.454	**<0.0001**	0.441	0.040	10.990	**<0.0001**
EMN200−EMN25	0.061	0.027	2.243	0.100	−0.019	0.021	−0.921	1.000
EMN200−SPEECH0	0.061	0.023	2.676	**0.037**	−0.031	0.018	−1.723	0.424
SPEECH0−NOISE	−0.042	0.026	−1.640	0.303	0.013	0.020	0.671	1.000
EMN25−NOISE	−0.042	0.030	−1.411	0.316	0.001	0.022	0.040	1.000
EMN200−NOISE	0.019	0.028	0.685	0.494	−0.018	0.021	−0.861	1.000

*P-*values are based on asymptotic Wald tests and are adjusted for multiple comparisons within family (the set of six contrasts, evaluated separately for *3SYL* and *SENT*) using the Bonferroni-Holm method. The Δ probability columns represent estimated arithmetic mean differences in per trial error probability (transformed from log-odds) between conditions. Bold values indicate an adjusted *p* < 0.05.

Across both stimulus types, no differences were observed in error rate between any of the tested conditions (SPEECH0, EMN25, and EMN200) vs. NOISE.

The generally high listener agreement and lack of strong differences in inter-rater reliability between experimental conditions support our approach to flagging errorful trials, which required that *all* listeners annotated an error. While we did not expect this choice to impact overall results, we also analyzed error rate using a different analytic choice, marking trials as errors if *any* listeners annotated an error. To avoid a singular fit, the random effects structure of those models needed to be simplified, removing the random slope for delay. The results of that analysis are included in Supplementary Tables S1, S2, which show very similar effects as shown in Tables 5, 6, with the exception that the EMN200 vs. SPEECH0 contrast is no longer significant for *3SYL* in this formulation.

### 3.4 Fundamental frequency

Praat-based estimates of mean *F_0_* and *F_0_* modulation (standard deviation within trial) were extracted from all trials. Figure 5 shows box plots of mean *F_0_* (top) and *F_0_* modulation (bottom) by speaker across conditions for *3SYL* (left) and *SENT* (right) stimuli.

**Figure 5:**
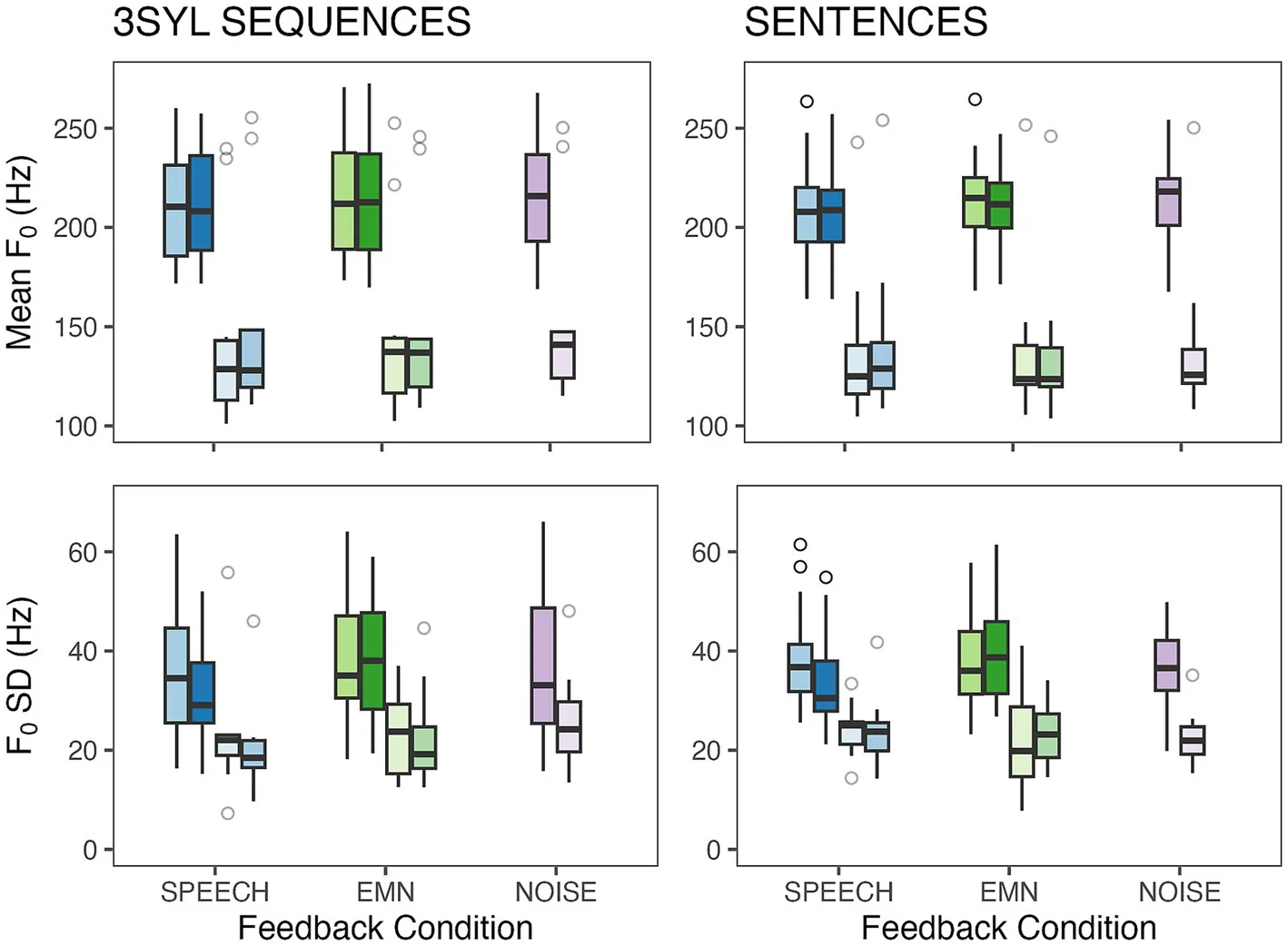
Mean F0 (top row) and mean standard deviation of F0 (bottom row) across experimental manipulations. Left: Results from 3-syllable sequence stimuli. Right: Results from sentence stimuli. Because of vocal pitch range differences by sex, women (higher F0 values, higher opacity) and men (lower F0 values, lower opacity) are shown separately. For each sex, darker shades (right boxes and whiskers in each pair) represent data from the 200 ms delay condition (SPEECH200, EMN200), while lighter shades (left boxes) represent data from the minimal delay conditions (SPEECH0, EMN25). Boxes span the interquartile (IQR) range; whiskers extend to 1.5 times the IQR, with circles indicating outlier participants.

Four linear mixed effects models were estimated (2 each for *3SYL* and *SENT* stimulus types, fit to *F_0_* and *F_0_* modulation). Supplementary Tables S3–S6 provide detailed results of the model coefficient and planned contrast estimates. As expected, all models showed significant reductions in both mean *F_0_* and *F_0_* modulation for men in comparison to women. Models revealed significant interactions between condition (SPEECH / EMN) and delay (minimal / 200 ms) for both *F_0_* and *F_0_* modulation for both stimulus types.

Evaluating within-condition effects of delay on *F_0_* using *emmeans*, while correcting for family-wise error rate, showed no significant effects for SPEECH200 – SPEECH0 or for EMN200 – EMN25 for either stimulus type. Subthreshold differences in the sign of the changes (*F_0_* increased on average with delay for SPEECH and decreased with delay for EMN), however, supported the observed interactions. Further, F0 was significantly higher for EMN200 vs. SPEECH0 for *3SYL* stimuli [estimate = +2.676 Hz, *t*(70.2) = 3.739, *p_adj_* = 0.002] though this difference was not significant for sentences.

Pairwise comparisons showed that, while controlling for other covariates, speakers reduced their *F_0_* modulation (lower standard deviation within trials) for both *3SYL* [estimate = −3.547 Hz, *t*(93.6) *= −*4.360, *p_adj_* = 0.0002] and *SENT* [estimate = −3.620 Hz, *t*(132.0) = −4.012, *p_adj_* = 0.0006] for SPEECH200 vs. SPEECH0 feedback. Conversely, there was no significant change in *F_0_* modulation for EMN200 vs. EMN25 feedback for either *3SYL* [estimate = +0.873 Hz, *t*(174.4) *=* 0.916, *p_adj_* = 0.8833] or *SENT* stimuli [estimate = +1.659, *t*(244.0) *=* 1.571, *p_adj_* = 0.3521].

Compared with the constant NOISE condition, *F_0_* was significantly lower in SPEECH0 and EMN200 conditions for both stimulus types. *F_0_* modulation was also significantly lower in SPEECH0 compared to NOISE for 3SYL stimuli only.

### 3.5 Intensity

Praat-based estimates of vocal intensity and intensity modulation (standard deviation within trial) were extracted from all trials. Figure 6 shows box plots of mean intensity (top) and intensity modulation (bottom) by speaker across conditions for *3SYL* (left) and *SENT* (right) stimuli. As with fundamental frequency, 4 linear mixed effects models were estimated (2 each for *3SYL* and *SENT* stimulus types). Supplementary Tables S7–S10 provide detailed results of the model coefficient and planned contrast estimates.

**Figure 6:**
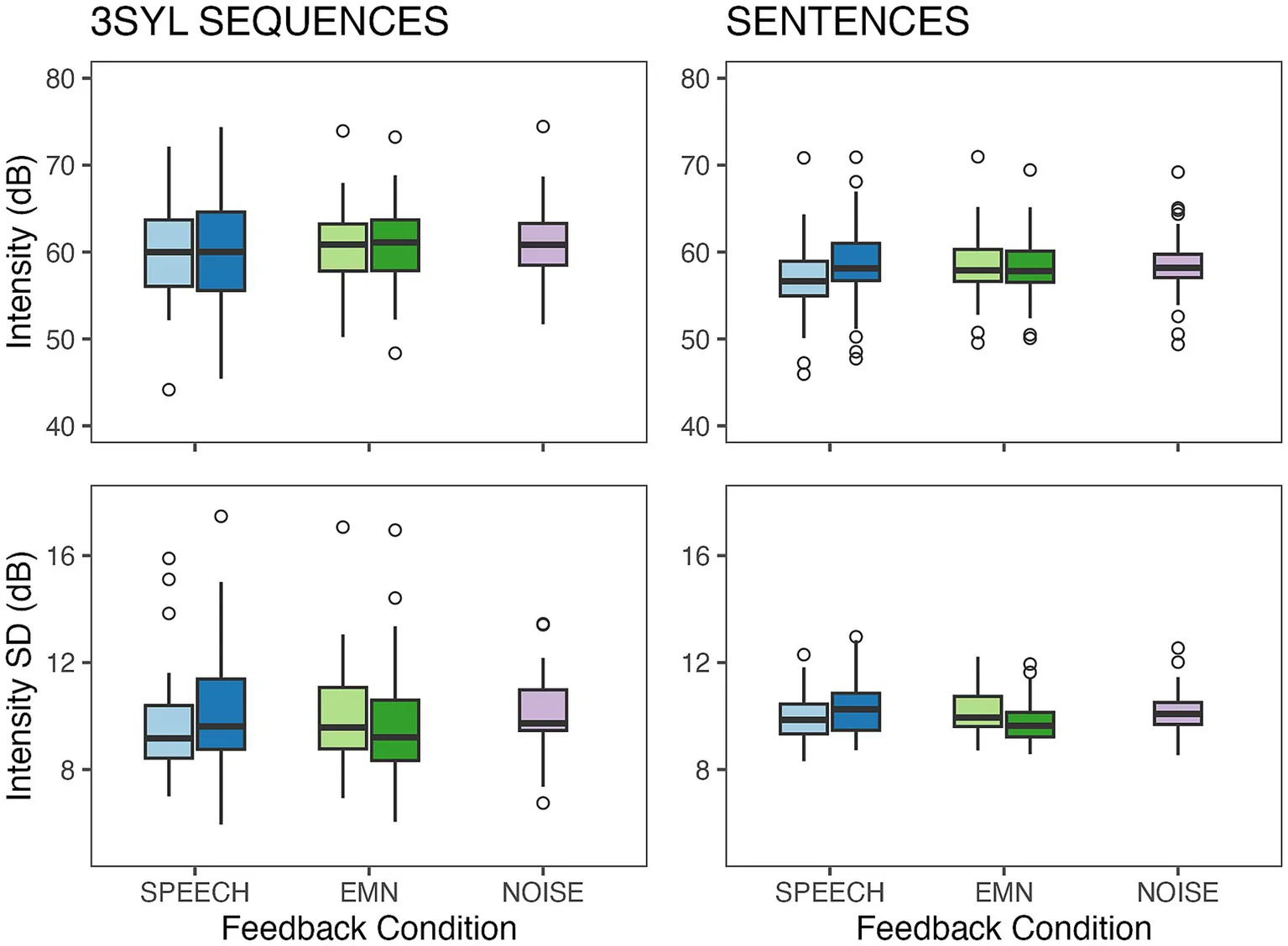
Mean intensity in decibels (top row) and mean standard deviation of intensity in decibels (bottom row) across experimental manipulations. Note that levels are uncalibrated, so absolute intensities are not meaningful. Left: Results from 3-syllable sequence stimuli. Right: Results from sentence stimuli. Darker shades (right boxes and whiskers in each pair) represent data from the 200 ms delay condition (SPEECH200, EMN200), while lighter shades (left boxes) represent data from the minimal delay conditions (SPEECH0, EMN25). Boxes span the interquartile (IQR) range; whiskers extend to 1.5 times the IQR, with circles indicating outlier participants.

Statistically significant interactions were observed between condition (SPEECH / EMN) and delay (minimal/200 ms) on both mean intensity and intensity modulation, for both *3SYL* and *SENT* stimuli. Planned comparisons, controlling for other covariates and for family-wise error rate, showed significantly increased intensity levels for SPEECH200 vs. SPEECH0 for *SENT* stimuli (estimate = +1.52 dB, *t*(69.7) *= 8.446*, *p_adj_* < 0.0001), with a trend in the same direction for *3SYL* stimuli that was not significant after correcting for multiple comparisons [estimate = +0.485 dB, *t*(58.4) = 2.447, *p_adj_* = 0.070]. No significant changes in intensity were observed for EMN200 compared to EMN25. Notably, the EMN200 condition resulted in significantly higher speech intensity levels compared to SPEECH0 [*3SYL*: estimate = +0.993 dB, *t*(58.5) = 5.003, *p_adj_* < 0.001; *SENT*: estimate = +1.302 dB, *t*(68.4) = 7.283, *p_adj_* < 0.001], as did the NOISE condition [SPEECH0 – NOISE *3SYL* estimate = −1.247 dB, *t*(4477.1) = −8.035, *p_adj_* < 0.0001; *SENT* estimate = −1.575 dB, *t*(3642.0) = −9.804, *p_adj_* < 0.0001]. Although it was not a planned contrast, the EMN25-SPEECH0 model coefficient was also significant (uncorrected) for both stimulus types. These results converge on the idea that exposure to any conditions involving speech-shaped noise results in increased speech intensity relative to typical feedback conditions.

Intensity modulation was significantly *increased* in the SPEECH200 vs. SPEECH0 condition for both *3SYL* [estimate = +0.590 dB, *t*(52.9) *=* 4.373, *p_adj_* = 0.0003] and *SENT* stimuli [estimate = +0.401 dB, *t*(76.0) *=* 4.111, *p* = 0.0006]. A small, statistically significant *decrease* in intensity modulation was seen for EMN200 vs. EMN25 for *SENT* stimuli [estimate = −0.281 dB, *t*(116.2) *= −2*.588, *p_adj_ =* 0.0436], with no difference for *3SYL* stimuli (estimate = −0.229 dB, *t =* −1.574, *p_adj_* = 0.2398). The NOISE condition elicited increased intensity modulation compared to SPEECH0, EMN25, and EMN200 conditions for *3SYL* stimuli. For sentence stimuli, increased intensity modulation was only significant for the EMN200 – NOISE contrast.

## 4 Discussion

The results of this study reveal marked differences in the effects of delayed speech (SPEECH200) vs. delayed envelope modulated noise (EMN200) feedback across the set of segmental and suprasegmental outcome measures. This is despite the fact that the SPEECH200 and EMN200 feedback conditions have virtually identical rhythmic envelope structure. For all six dependent variables, across both syllable sequence and sentence productions, we observed a statistically significant interaction between condition (SPEECH/EMN) and delay (minimal/200 ms), supporting our central hypothesis that delayed EMN would not disrupt speech in the same ways as canonical DAF. The results were striking for the two primary outcome measures: speech rate and error rate. Compared to SPEECH0 (typical feedback), SPEECH200 led to large speaking rate reductions (−0.776 syllables/s for *3SYL* and −0.887 syllables/s for *SENT* stimuli) and large increases in the probability of an error (~31% increase for *3SYL* and ~44% increase for *SENT* stimuli). Conversely, compared to EMN25 (minimally delayed envelope modulated noise), EMN200 resulted in very slight but significant *increases* in speaking rate for both stimulus types and much smaller changes in error probability that were not statistically significant (though note that there was a ~ 6% increase in errors for EMN200 vs. SPEECH0 for *3SYL*). Thus, the findings of this study strongly argue that the displacement of the speech envelope alone does not account for the disruptions of speech output elicited by canonical DAF.

Suprasegmental measures showed an overall similar pattern across the measured variables: *F_0_* and *F_0_* modulation, and intensity and intensity modulation, each also revealing statistically significant interactions between condition and delay for both stimulus types. The observed within-condition changes in these secondary measures were small and somewhat varied and should thus be interpreted with caution. However, the changes we observed with delay were more consistent within the SPEECH condition. Canonical DAF—controlling for age, sex, and trial number – elicited significant reductions in *F_0_* modulation (e.g., more monotonous speech), while EMN200 (vs. EMN25) showed no significant effects. The SPEECH200 condition (vs. SPEECH0) also elicited small increases in vocal intensity (significant only for sentences) and intensity modulation (significant across both stimulus types), while no differences in intensity were observed as a function of delay for EMN. Interestingly, a significant *decrease* in intensity modulation was observed for EMN200 (vs. EMN25) for sentences only, though it should be noted this effect was very small and only marginally significant, even without a secondary adjustment to control false positives across models and dependent measures. Thus, as with the segmental/articulatory variables, we observed that delayed envelope modulated noise did not have the same or similar effects as delayed speech feedback. These results further argue that, while the timing of the rhythmic envelope of the auditory feedback signal may be important, the phonetic and other spectral content only available in the SPEECH feedback signals appear to be critical in driving speech disruptions under DAF.

### 4.1 The displaced rhythm hypothesis

The observed pattern of results is inconsistent with the strong claims of the displaced rhythm hypothesis (Howell et al., 1983), which would predict that delayed EMN should be *as disruptive* to speech output as delayed SPEECH. This hypothesis would predict that EMN200, which has the same rhythm / envelope contour as speech-based feedback, would elicit speech rate reductions and increases in speech errors similar to SPEECH200; however, our results show much more limited effects when the speech-specific content is replaced with aperiodic speech-shaped broadband noise. The DRH argues that the central reason that DAF interferes with speech is because it involves producing one utterance while hearing an auditory signal that is out of synchrony/phase with what is expected. This theoretical perspective is also broadly consistent with the view that rhythmic motor behavior emerges from coupled motor (action) and sensory (perception) systems, organized around relative phase relationships (Kelso, 1995). While there is myriad evidence indicating such action-perception coupling, Howell’s DRH more strongly argues that the rhythmic changes are *all that matter*, and that phonemic content is not relevant to the speech disruptions seen under canonical DAF.

The present results do show some modest evidence that delayed EMN disrupts speech (e.g., increased error rate for *3SYL* stimuli compared to minimally-delayed speech feedback) but primarily point to the importance of the *speech-specific content* of the feedback signal above and beyond the effects of the displaced rhythm. It is important to note, however, that some previous results have provided specific support for the DRH (Howell et al., 1983; Howell and Archer, 1984; Howell and Powell, 1987; Kaspar and Rübeling, 2011), though those most directly comparable to this work (e.g., that intentionally removed speech-specific content) were obtained in quite small samples (*N* = 5 in Howell et al., 1983; *N* = 10 female participants in Howell and Archer, 1984) compared to the present study (*N* = 38).

There are notable differences between the feedback signals used in these original studies and the present work. Our approach removes phonetic content while also removing periodicity and temporal fine structure present in the speech signal. The approaches used by Howell and colleagues also did not retain speech-specific temporal fine structure but either preserved periodicity from the speech signal (Howell et al., 1983) or replaced the amplitude-modulated carrier with a periodic, complex tone (Howell and Archer, 1984). Thus, we cannot conclude that the phonetic detail of specific speech sounds must be preserved for DAF to cause large speech disruptions, but our data indicate that some speech-specific properties, which might include underlying periodicity, are necessary.

The study by Kaspar and Rübeling (2011) had a large sample size (*N* = 151) and showed that reading time (inversely related to speech rate) when producing repeated syllable tuples (/ta/ /ta/ and /ta/ /li/) was increased at 200 ms delays, but that the increase in reading time was greater when one syllable was accentuated (creating a different rhythmic and perhaps pitch envelope) than when neither syllable was accentuated. This difference occurred irrespective of the phonemic content manipulation, providing evidence that the displaced rhythm was *especially critical* in slowing speech output. As these authors acknowledge, however, it was not possible to fully disentangle rhythm and content in this study design, in which the auditory feedback signal always included speech content. Our approach to *eliminate* speech-specific spectral content from the auditory input signal while preserving the rhythmic envelope (also Howell et al., 1983; Howell and Archer, 1984) offers a more direct test of the specific importance of rhythm, and the results obtained here are inconsistent with the interpretation of Kaspar and Rübeling. One possible reason for the discrepancy is that by removing the speech-specific spectral content of the auditory signal (as in our EMN conditions), speakers process the auditory signal *differently*, perhaps by relying on distinct populations of neurons and pathways in the auditory cortex, or by reducing attention to the input. It is well established, for example, that passive listening to speech vs. matched non-speech signals of various forms elicit different activations across the auditory hierarchy (e.g., Binder et al., 2000; DeWitt and Rauschecker, 2012; Stoppelman et al., 2013). It is also possible that the irregularity inherent in our noise carrier makes the feedback signal less salient (easier to ignore) and/or influences whether the auditory system treats the feedback as self-produced speech.

### 4.2 The hypothesized interplay of displaced rhythms and content

Another possible explanation of the current results is that *both* rhythmic synchrony and phonetic and/or other speech-specific *content* are important to the mechanisms of DAF-induced speech disruptions, but that they operate through different mechanisms. This view is consistent with that of Pfordresher and colleagues (Pfordresher, 2006; Pfordresher and Benitez, 2007), who developed a theoretical framework for understanding delayed auditory feedback in speech and music performance, with dissociable effects related to rhythmic asynchrony and altered content. Because disruption of speech and music performance is not limited to asynchronies in the auditory feedback signal, Pfordresher (2006) described the DRH as “too narrow,” arguing for a need to also account for the content of the auditory feedback signal.

Malloy et al. (2022) also take this point of view, describing a theoretical model that builds on the GODIVA model of speech sequence production (Bohland et al., 2010), proposing differential roles for rhythm/asynchrony and for the content of auditory feedback. In this model, the motor system entrains local rhythms in the auditory cortex (via neural *antiphase* coupling) in anticipation of the incoming speech envelope. The phenomenon known as speech-induced suppression (SIS; Numminen et al., 1999; Curio et al., 2000; Houde et al., 2002) has demonstrated that the auditory cortical evoked response to self-produced speech is *suppressed* relative to passive listening; thus, we hypothesize that the entrainment of speech auditory cortex during running speech allows for peaks in the envelope of self-produced speech (with typical feedback latencies) to arrive during the troughs of the entrained auditory rhythms (time windows of *hypoexcitability*). In this way, when speech is regularly timed, reduced auditory excitability helps to prevent interference from the incoming feedback signal. The disruptive effects of DAF, then, are viewed as owing to peaks in the envelope of the feedback signal (i.e., larger amplitude auditory inputs) arriving at time intervals of unexpected *hyperexcitability* because of the mismatch between the entrained expectations and the delayed arrival of feedback. In this model, the specific *content* of the speech signal then determines any interfering effects, driving changes to the neural representations comprising the speech plan, specifically due to the hyperexcitable auditory state. Our hypothesis is that, even when high amplitude auditory inputs arrive at times of hyperexcitability due to delays, if they are not speech-like (as in the delayed EMN condition), they will not disrupt the speech plan. This model is largely consistent with invasive electrophysiological data in humans showing that many of the same neural populations that show strong SIS also show large responses to speech under DAF (Ozker et al., 2024), though this study did not specifically investigate cortical rhythmic activity.

In sum, speech disruptions occur in this speculative model both because of the displaced rhythm *and* because of the specific speech content arriving at unexpected time intervals. The reduced disruptions observed during the EMN200 condition, then, are easily explained as lacking this second critical mechanism; because the feedback signal contains only speech-shaped noise that does not activate speech-specific neural representations, it cannot directly interfere with the ongoing speech plan. It is important to note that this model remains largely untested, and testing may prove difficult due to limitations of experimental methods for measuring local field potentials and cortical rhythms during running speech. It also cannot currently distinguish between the proposed importance of speech-specific content vs. generally reduced attention to the feedback signal that may occur as it becomes less obviously tied to self-generated actions by the speaker.

### 4.3 Effects of auditory feedback alterations on suprasegmental measures

Many previous studies have reported suprasegmental speech alterations due to DAF. In particular, studies often report increased speech intensity (e.g., Fairbanks, 1955; Siegel et al., 1982; Howell and Archer, 1984; Chesters et al., 2015; Ozker and Hagoort, 2025) with less consistent findings about voice fundamental frequency (Lechner, 1979; Siegel et al., 1982; Chesters et al., 2015; Ozker and Hagoort, 2025). Howell and Powell (1987) wrote that “subjects often report that when they hear DAF, they raise their voice levels to increase the amount of feedback that is not delayed; this feedback arrives by bone conduction, leakage from the headphones, and so forth” (p. 167). Reports of increased *F_0_* might have similar explanations, with the speaker attempting to hear their true speech more clearly. Reports of monotone speech (Howell, 2004), or a general flattening of prosodic variation, are perhaps more interesting and with less clear explanation, though far less commonly reported.

In this study, we observed interactions between delay and condition for all four suprasegmental variables, suggesting that delayed SPEECH and delayed EMN had different effects. Looking more closely, the within-condition comparisons revealed effects that were small and somewhat inconsistent. Small intensity increases occurred (relative to SPEECH0) for the *SENT* (+1.52 dB) stimuli (with a trend for a small increase in *3SYL* stimuli that was not significant), whereas no intensity changes were seen for EMN. Somewhat surprisingly, we saw no significant effects on mean *F_0_* for SPEECH200 compared to SPEECH0 feedback or for EMN200 compared to EMN25 feedback, though the directions of sub-threshold changes differed for the two feedback types. Our data partially supported the idea of flattening of prosody under DAF. Compared to SPEECH0, SPEECH200 feedback resulted in significantly reduced *F_0_* modulation, increased mean intensity, and increased intensity modulation. The decreased *F_0_* modulation is in agreement with studies showing decreased pitch modulation under DAF (Fairbanks, 1955; Chesters et al., 2015). However, we saw *increases* in intensity modulation for the SPEECH200 condition (both stimulus types), which was surprising given the *decreases* in *F_0_* modulation, and the fact that vocal pitch and intensity tend to be correlated. The EMN200 had the opposite effect on intensity modulation, showing significantly decreased modulation for the *SENT* stimuli. The increased intensity modulation under canonical DAF is a novel finding; it may be a result of overall increases in loudness during DAF (also seen here), or it may reflect attempts to compensate for the mismatched amplitude contour in the feedback signal. The fact that EMN shows the opposite effect further demonstrates that asynchronous rhythms cannot fully explain the speech changes observed under DAF. This is true for both segmental and suprasegmental features.

### 4.4 Comparisons with constant noise masking

The experiment included an additional feedback condition (NOISE) that used the same speech-shaped noise that served as the carrier for EMN but had approximately constant amplitude throughout each trial (see Figure 2). This condition served as a control to determine how aperiodic noise feedback—without the imposition of any specific envelope or delay—impacted the outcome variables. This helped us to determine if any effects of EMN were likely related to it being treated as a form of masking noise.

We observed an increased speech rate for EMN200 vs. EMN25 (and vs. SPEECH0 for the *SENT* condition only). In the case of sentences, this paralleled a rate increase for the NOISE condition. Further, there were no differences in rate between the EMN200 and NOISE conditions for either stimulus type. This congruence is suggestive of delayed envelope modulated noise acting as a noise masker. However, the similarities were less clear in the case of *3SYL* stimuli, where there was a significant rate increase for EMN200 vs. EMN25, and a much smaller, non-significant change for NOISE vs. EMN25. For error rate, NOISE had no significant effects. In examining the suprasegmental features, NOISE caused a significant increase in intensity for both stimulus types (vs. SPEECH0), which was mirrored by a slightly smaller but significant increase for EMN200 vs. SPEECH0 (and the difference between EMN200 and NOISE was not significant for either stimulus type). NOISE also caused a significant increase in *F_0_* compared to SPEECH0 for both stimulus types, whereas EMN200 revealed lesser changes that were only significant for *3SYL.* Further, there were significant differences in *F_0_* for the EMN200 vs. NOISE comparison for both stimulus types, which indicates that the two signals were not being treated equivalently. A similar, significant difference between EMN200 and NOISE was observed for intensity modulation, for both stimulus types.

Taken together, these findings provide some evidence that EMN200 may have been treated qualitatively similarly to the constant masking noise (with most effects at least in the same direction). However, there is also evidence, as noted above, that the effects are not identical. Future work is necessary to determine to what extent the effects we observed under EMN200 were due to a partial masking effect versus being driven specifically by the delayed rhythmic envelope.

### 4.5 Differences from previous studies

There are many differences in the present study compared to previous work aimed at testing the influence of asynchronous rhythms between speech motor outputs and auditory inputs. Most notably, previous studies (Howell et al., 1983; Howell and Archer, 1984; Kaspar and Rübeling, 2011) have focused predominantly on “performance time” needed to recite a prescribed set of simple speech materials (e.g., a randomized list of 40 vowels in Howell and Archer, 1984; or a sequence of 6 syllable dyads in Kaspar and Rübeling, 2011). In our study, speech rate was one of several dependent variables, though arguably not the most important one. We were also interested in how altered feedback manipulations drive speech errors, as rate reductions can be seen as a compensatory strategy (e.g., extending vowel durations to allow time for feedback to match expectations) aimed at reducing errors (speed-accuracy tradeoff). In our study, participants were encouraged to maintain specific speaking rates. For the *3SYL* task, the target rate was 5 syllables per second, which was indicated by a “visual metronome” signal. Previous work in different participants (Malloy et al., 2022) showed that the visual metronome resulted in smaller rate reductions under DAF than other published studies, but participants still slowed their speech (also seen here for SPEECH200 vs. SPEECH0). For the *SENT* repetition task, a visual “progress bar” moved from left to right during the production period, pacing the overall sentence production. These visual pacing signals were present for both the SPEECH and EMN conditions and could be one reason why speech rate reductions were not observed for EMN200 vs. EMN25 (in fact, slightly increased rates were seen for the EMN200 conditions). Follow-up work could help determine what, if any influence, these pacing signals had on the outcome measures, though it is unlikely they acted in fundamentally different ways between SPEECH and EMN feedback.

A second key difference, as previously discussed, was that the spectral content of the feedback signal in our EMN conditions was replaced by speech-shaped noise (SSN), which was modulated by the speech envelope, extracted in near real-time. In Howell and Archer (1984), a similar approach was used, but rather than using SSN, a 500-Hz square wave (complex tone) was multiplied by a derived envelope signal. Interestingly, this would result in a signal with a clear, consistent pitch, whereas our SSN signal was aperiodic. Our signal also removed temporal fine structure: the rapid waveform variations that, in part, carry phonetic identity in speech. While the 500 Hz complex tone used by Howell and Archer carried its own fine structure, it differed from that found in the original speech signal and thus is less likely to be the cause of different outcomes. It is also notable that speech is not produced with a constant pitch or with continuous voicing, and a 500 Hz fundamental frequency would be considerably higher than the vocal pitch of most humans, including children. Therefore, it is not obvious that Howell and Archer’s periodic feedback signal was particularly speech-like. Future work should replicate those long-standing results in a larger sample and work to determine the specific characteristics of the delayed feedback signal that are necessary to create large disruptive effects.

Finally, our study used different speech materials, which were more varied than similar previous studies. The use of both *3SYL* and *SENT* stimuli allowed us to observe generalized effects of DAF that occurred regardless of lexical-semantic context or the presumed differences in speech planning load across conditions. We found that SPEECH200 had consistent effects for most outcome variables across the two stimulus types, suggesting that disruptions to speech occur at a relatively low level, likely not related to linguistic processing. Our study, while having a relatively large sample size, also notably included a large amount of data per participant (120 *3SYL* productions and 98 *SENT* productions across feedback conditions per participant). The large samples likely helped to stabilize mixed-effects statistical models that included random by-participant intercepts and random by-participant slopes for delay.

### 4.6 Limitations

Though our results provide a clear, objective comparison of the effects of delayed SPEECH and delayed EMN feedback on speech motor output, this study has several limitations. First, a latency of 25 ms was used in the EMN25 condition, while, in the congruent minimum latency speech feedback condition (SPEECH0), the latency was only ~12 ms. Despite these differences, it has been previously shown that 25 ms delays resulted in no significant changes to speech rate and no significant increase in speech disfluencies (Stuart et al., 2002; Kalinowski et al., 1996, p. 199). Further, Veillette et al. (2025) tested participants’ thresholds for detecting auditory-motor asynchronies (>50% accuracy), with mean values of 59.8 ms prior to extended exposure to DAF. Thus, it is unlikely that participants’ speech was disrupted specifically by this short latency.

A few methodological choices also create limitations. The amplitude/intensity values in our audio recordings were not specifically calibrated, and thus it is not possible to recover absolute sound pressure levels of speech or speech feedback. Still, relative values (within the same speaker and session) are meaningful and point to generally expected results for the SPEECH conditions. Additionally, the SPEECH and EMN conditions were not mixed within the same runs but rather occurred in different experimental blocks within the same session. We cannot be certain that results would be the same if trials were mixed, but because the orders of block types were counterbalanced across participants, we are confident that differences between SPEECH and EMN feedback were not driven by order effects or learning throughout the session. Additionally, the SPEECH blocks (but not the EMN/NOISE blocks) also contained trials with 150 and 250 ms delays that were not included in these analyses. We cannot rule out that the lower predictability of delay conditions in the SPEECH blocks contributed to the larger observed changes in the outcome measures.

If participants treated the EMN conditions as different from natural, self-generated speech (e.g., as external signals), they may have also more readily habituated to it or learned not to attend to it during those blocks. Our design was unable to control for any differences in the allocation of attention across conditions, and thus differences in auditory attention could provide an alternative explanation to why EMN was less disruptive. Future work should consider interleaving EMN with SPEECH feedback to reduce attentional effects and/or concurrently measuring signals such as pupillometry to provide at least indirect measures of attentional allocation and mental effort.

Finally, while we attempted to recruit a representative sample from the greater Pittsburgh area, it is notable that only 10 of 38 participants reported their biological sex as male. While some previous studies have reported sex differences in susceptibility to DAF (Bachrach, 1964; Corey and Cuddapah, 2008; Chon et al., 2013; Stuart and Kalinowski, 2015), such findings are inconsistent across studies.

## 5 Conclusion

In this study, like previous ones using DAF, we observed that delaying speech signals by 200 ms elicited large, consistent disruptions across both articulatory and prosodic variables, and for both non-speech syllable sequences and sentences. On the other hand, envelope modulated noise, with the same overall rhythm as speech and the same 200 ms delay, produced relatively minimal disruptions. These findings, combined with earlier related work, suggest that the presence of speech-related spectral content in the auditory feedback signal is necessary to elicit the full disruptive effects of DAF, contrary to the displaced rhythm hypothesis. While motor-auditory asynchrony is important (and present in the disruptive SPEECH200 condition), the smaller effects of EMN observed here suggest that the specific spectral content of the feedback signal has an important role in speech disruptions under DAF. While more work is needed to distinguish between alternative explanations, our results are consistent with models that propose dissociable mechanisms for rhythmic asynchrony and feedback content in speech motor control. Future studies should focus on defining the specific characteristics of the feedback signal that are necessary to cause interference effects and on elucidating the underlying neural processes that lead to speech disruptions under DAF.

## Data Availability

Minimally preprocessed raw data and source code supporting the analyses and conclusions of this article will be made available upon publication via the Open Science Framework (https://doi.org/10.17605/OSF.IO/VZ6UH).

## Footnotes

1 While some studies have found increases in mean voice fundamental frequency, others have found no difference (Taitelbaum-Swead et al., 2019) or decreases under canonical DAF (e.g., Chesters et al., 2015; Kanty and Staroniewicz, 2025). F0 modulation is less often reported.

2
http://audacityteam.org

3 The TIMIT audio files were sampled at 16 kHz, limiting the upper end of the power spectrum to 8 kHz.

4 A 25 ms latency was chosen due to the additional processing requirements of the EMN condition; during pilot testing, shorter specified latencies sometimes resulted in discontinuous “choppy” feedback or other unexpected sound output.

5 Three of the eleven listeners completed a large fraction of the listening assignments (4,255 of 5,911 3SYL listening assignments and 3,602 of 4,796 SENT listening assignments). Note the number of listening assignments includes overlap between listeners and thus exceeds the number of experimental trials analyzed. All listeners were trained by author AO through video examples and a “pre-test” containing a specific set of trials from one participant (across all tasks).

6 The specific intensity levels extracted are uncalibrated, thus absolute levels are not meaningful; however, relative intensity differences within-subject are still meaningful as participants maintained the same microphone distance throughout the session.
