# Supplementary material for: The effects of delayed auditory feedback depend on both the rhythm and content of the feedback signal
Source: Front Hum Neurosci. 2026 Sep 16;20:1889468. doi: 10.3389/fnhum.2026.1889468 (PMC13623862; doi:10.3389/fnhum.2026.1889468)
Supplement: Supplementary file 1 [file Data_Sheet_1.PDF]

**Supplementary Table 1:** Alternative results from generalized linear mixed effects model estimates for error rate (compare to Table 5 in the main text). These models treated any trial that was annotated as containing at least one error by *any* listener as an error trial (cf. the analysis in the main text, which required agreement across all listeners). Separate models were fit for *3SYL* and for *SENT.* To avoid singular fits, the random slope for delay was dropped in both models. Reported *p*-values for individual fixed effects coefficients are obtained based on asymptotic Wald tests and are not adjusted for multiple comparisons. Model formula: *error ~ condition*delay + sex + age + trial + (1 | subject*).

| | 3SYL | | | | SENT | | | |
|---|---|---|---|---|---|---|---|---|
| *Fixed Effects* | *OR* | *CI* | *z* | *p* | *OR* | *CI* | *z* | *p* |
| intercept [SPEECH0] | 0.383 | 0.280 – 0.526 | -5.947 | **<0.001** | 0.096 | 0.061 – 0.152 | -10.071 | **<0.001** |
| condition [EMN25 vs. SPEECH0] | 1.045 | 0.834 – 1.310 | 0.386 | 0.700 | 0.909 | 0.655 – 1.262 | -0.567 | 0.570 |
| condition [NOISE vs. SPEECH0] | 1.097 | 0.877 – 1.374 | 0.811 | 0.418 | 0.928 | 0.664 – 1.297 | -0.438 | 0.661 |
| delay [SPEECH200 vs. SPEECH0] | 3.663 | 3.045 – 4.406 | 13.771 | **<0.001** | 10.82 | 8.394 – 13.938 | 18.407 | **<0.001** |
| sex [MALE - FEMALE] | 1.922 | 1.086 – 3.400 | 2.244 | **0.025** | 3.447 | 1.543 – 7.699 | 3.018 | **0.003** |
| age (centered) | 0.988 | 0.965 – 1.011 | -1.042 | 0.297 | 0.999 | 0.967 – 1.032 | -0.047 | 0.962 |
| trial (centered) | 1.001 | 0.997 – 1.005 | 0.623 | 0.534 | 0.999 | 0.993 – 1.006 | -0.251 | 0.802 |
| condition x delay [EMN200 vs. EMN25] vs. [SPEECH200 vs. SPEECH0] | 0.318 | 0.238 – 0.426 | -7.73 | **<0.001** | 0.101 | 0.067 – 0.152 | -10.915 | **<0.001** |
| | | | | | | | | |
| *Random Effects* | | | | *Variance* | | | | *Variance* |
| σ2 | | | | 3.290 | | | | 3.290 |
| τ00 SUBJECT | | | | 0.584 | | | | 1.161 |
| ICC | | | | 0.151 | | | | 0.261 |
| Marginal R2 | | | | 0.090 | | | | 0.236 |
| Conditional R2 | | | | 0.227 | | | | 0.435 |

**Supplementary Table 2:** Results of planned contrasts for error rate, following Supplementary Table 1 (compare to Table 6 in the main text). As above, any trial that was annotated as containing at least one error by *any* listener was used as an error trial in this supplemental analysis. *P*-values are based on asymptotic Wald tests and are adjusted for multiple comparisons within family (the set of six contrasts, evaluated separately for *3SYL* and *SENT*) using the Bonferroni-Holm method. The Δ probability columns represent estimated arithmetic mean differences in per trial error probability (transformed from log-odds) between conditions.

| | *3SYL* | | | | *SENT* | | | |
|---|---|---|---|---|---|---|---|---|
| *Contrast* | Δ *Probability* | *SE* | *z* | $p_{adj}$ | Δ *Probability* | *SE* | *z* | $p_{adj}$ |
| SPEECH200 - SPEECH0 | 0.3136 | 0.0213 | 14.756 | **<0.0001** | 0.5074 | 0.0293 | 17.296 | **<0.0001** |
| EMN200 - EMN25 | 0.0361 | 0.0266 | 1.355 | 0.702 | 0.0107 | 0.0204 | 0.525 | 1.000 |
| EMN200 - SPEECH0 | 0.0462 | 0.0218 | 2.119 | 0.170 | -0.0011 | 0.0176 | -0.062 | 1.000 |
| SPEECH0 - NOISE | -0.0214 | 0.0265 | -0.807 | 1.000 | 0.0094 | 0.0213 | 0.441 | 1.000 |
| EMN25 - NOISE | -0.0112 | 0.0306 | -0.367 | 1.000 | -0.0024 | 0.0237 | -0.102 | 1.000 |
| EMN200 - NOISE | 0.0248 | 0.0267 | 0.930 | 1.000 | 0.0083 | 0.0211 | 0.393 | 1.000 |

**Supplementary Table 3:** Results from linear mixed effects model estimates for fundamental frequency ($F_0$). Separate models were fit for *3SYL* and for *SENT.* Separate models were fit for *3SYL* and for *SENT.* Reported *p*-values for individual fixed effects coefficients are not adjusted for multiple comparisons. Model formula: *F0 ~ condition*delay + sex + age + trial + (1 + delay | subject*).

| | **3SYL** | | | | | **SENT** | | | | |
|---|---|---|---|---|---|---|---|---|---|---|
| *Fixed Effects* | *Estimates* | *CI* | *t* | *df* | *p* | *Estimates* | *CI* | *t* | *df* | *p* |
| Intercept | 211.757 | 198.372 – 225.142 | 32.105 | 35.393 | **<0.001** | 207.814 | 197.478 – 218.149 | 40.789 | 35.781 | **<0.001** |
| condition [EMN25 - SPEECH0] | 3.702 | 2.433 – 4.972 | 5.716 | 4477.065 | **<0.001** | 3.171 | 1.755 – 4.587 | 4.389 | 3641.102 | **<0.001** |
| condition [NOISE - SPEECH0] | 5.247 | 3.977 – 6.518 | 8.099 | 4477.094 | **<0.001** | 4.089 | 2.636 – 5.542 | 5.516 | 3641.059 | **<0.001** |
| delay [SPEECH200 - SPEECH0] | 0.873 | -0.553 – 2.300 | 1.221 | 70.004 | 0.226 | 0.918 | -0.532 – 2.368 | 1.258 | 87.765 | 0.212 |
| sex [MALE - FEMALE] | -68.799 | -94.676 – -42.923 | -5.398 | 34.995 | **<0.001** | -68.418 | -88.185 – -48.651 | -7.027 | 34.999 | **<0.001** |
| age (centered) | -0.352 | -1.395 – 0.690 | -0.686 | 34.993 | 0.497 | -0.465 | -1.262 – 0.331 | -1.186 | 35.002 | 0.244 |
| trial (centered) | 0.106 | 0.084 – 0.127 | 9.735 | 4504.710 | **<0.001** | 0.03 | 0.001 – 0.060 | 2.019 | 3668.72 | **0.044** |
| condition x delay [EMN200 - EMN25] - [SPEECH200 - SPEECH0] | -1.900 | -3.539 – -0.261 | -2.273 | 4477.020 | **0.023** | -2.886 | -4.726 – -1.047 | -3.076 | 3641.047 | **0.002** |
| | | | | | | | | | | |
| *Random Effects* | | | | | *Variance* | | | | | *Variance* |
| $\sigma^2$ | | | | | 159.201 | | | | | 166.896 |
| $\tau_{00}$ SUBJECT | | | | | 1220.354 | | | | | 729.925 |
| $\tau_{11}$ SUBJECT.delay200 | | | | | 8.816 | | | | | 6.303 |
| $\rho_{01}$ SUBJECT | | | | | 0.212 | | | | | -0.266 |
| ICC | | | | | 0.887 | | | | | 0.811 |
| Marginal R2 | | | | | 0.399 | | | | | 0.513 |
| Conditional R2 | | | | | 0.932 | | | | | 0.908 |

**Supplementary Table 4:** Results of planned contrasts for fundamental frequency ($F_0$). *P*-values are adjusted for multiple comparisons within family (the set of six contrasts, evaluated separately for *3SYL* and *SENT*) using the Bonferroni-Holm method.

| | ***3SYL*** | | | | | ***SENT*** | | | | |
|---|---|---|---|---|---|---|---|---|---|---|
| *Contrast* | *Estimate* | *SE* | *t* | *df* | *p* | *Estimate* | *SE* | *t* | *df* | *p* |
| SPEECH200 - SPEECH0 | 0.873 | 0.715 | 1.221 | 70.000 | 0.4120 | 0.918 | 0.73 | 1.258 | 87.8 | 0.4232 |
| EMN200 - EMN25 | -1.026 | 0.807 | -1.272 | 112.900 | 0.4120 | -1.968 | 0.824 | -2.387 | 142 | 0.0731 |
| EMN200 - SPEECH0 | 2.676 | 0.716 | 3.739 | 70.200 | **0.0019** | 1.203 | 0.725 | 1.659 | 85.6 | 0.3023 |
| SPEECH0 - NOISE | -5.247 | 0.648 | -8.099 | 4477.100 | **<0.0001** | -4.089 | 0.741 | -5.516 | 3641.1 | **<0.0001** |
| EMN25 - NOISE | -1.545 | 0.747 | -2.067 | 4477.000 | 0.1164 | -0.918 | 0.839 | -1.094 | 3641.1 | 0.4232 |
| EMN200 - NOISE | -2.571 | 0.807 | -3.187 | 112.900 | **0.0074** | -2.886 | 0.841 | -3.43 | 153.9 | **0.0039** |

**Supplementary Table 5:** Results from linear mixed effects model estimates for fundamental frequency modulation (within-trial standard deviation of $F_0$ in Hz). Separate models were fit for *3SYL* and for *SENT.* Separate models were fit for *3SYL* and for *SENT.* Reported *p*-values for individual fixed effects coefficients are not adjusted for multiple comparisons. Model formula: *F0sd ~ condition*delay + sex + age + trial + (1 + delay | subject*).

| | **3SYL** | | | | | **SENT** | | | | |
|---|---|---|---|---|---|---|---|---|---|---|
| *Fixed Effects* | *Estimates* | *CI* | *t* | *df* | *p* | *Estimates* | *CI* | *t* | *df* | *p* |
| intercept | 35.681 | 31.261 – 40.100 | 16.32 | 39.973 | **<0.001** | 38.085 | 35.231 – 40.939 | 26.904 | 43.461 | **<0.001** |
| condition [EMN25 - SPEECH0] | 1.937 | 0.252 – 3.622 | 2.25 | 4477.132 | **0.024** | -1.098 | -3.127 – 0.930 | -1.062 | 3641.165 | 0.288 |
| condition [NOISE - SPEECH0] | 2.977 | 1.292 – 4.662 | 3.46 | 4477.181 | **0.001** | -1.942 | -4.023 – 0.139 | -1.83 | 3641.092 | 0.067 |
| delay [SPEECH200 - SPEECH0] | -3.547 | -5.162 – -1.931 | -4.36 | 93.63 | **<0.001** | -3.616 | -5.398 – -1.833 | -4.012 | 131.939 | **<0.001** |
| sex [MALE - FEMALE] | -13.675 | -21.411 – -5.939 | -3.59 | 35.001 | **0.001** | -13.587 | -18.839 – -8.335 | -5.252 | 34.977 | **<0.001** |
| age (centered) | 0.177 | -0.135 – 0.489 | 1.15 | 35 | 0.257 | -0.026 | -0.238 – 0.185 | -0.254 | 34.991 | 0.801 |
| trial (centered) | 0.05 | 0.021 – 0.078 | 3.45 | 4513.192 | **0.001** | 0.013 | -0.030 – 0.055 | 0.581 | 3677.743 | 0.561 |
| condition x delay [EMN200 - EMN25] - [SPEECH200 - SPEECH0] | 4.42 | 2.245 – 6.594 | 3.99 | 4477.061 | **<0.001** | 5.274 | 2.640 – 7.908 | 3.926 | 3641.073 | **<0.001** |
| | | | | | | | | | | |
| *Random Effects* | | | | | *Variance* | | | | | *Variance* |
| σ2 | | | | | 280.240 | | | | | 342.167 |
| τ00 SUBJECT | | | | | 134.099 | | | | | 44.224 |
| τ11 SUBJECT.delay200 | | | | | 6.436 | | | | | 2.307 |
| ρ01 SUBJECT | | | | | -0.604 | | | | | 0.139 |
| ICC | | | | | 0.299 | | | | | 0.120 |
| Marginal R2 | | | | | 0.106 | | | | | 0.090 |
| Conditional R2 | | | | | 0.373 | | | | | 0.200 |

**Supplementary Table 6:** Results of planned contrasts for fundamental frequency modulation (Hz). *P*-values are adjusted for multiple comparisons within family (the set of six contrasts, evaluated separately for *3SYL* and *SENT*) using the Bonferroni-Holm method.

| | ***3SYL*** | | | | | ***SENT*** | | | | |
|---|---|---|---|---|---|---|---|---|---|---|
| *Contrast* | *Estimate* | *SE* | *t* | *df* | *p* | *Estimate* | *SE* | *t* | *df* | *p* |
| SPEECH200 - SPEECH0 | -3.547 | 0.813 | -4.360 | 93.6 | **0.0002** | -3.616 | 0.901 | -4.012 | 132.0 | **0.0006** |
| EMN200 - EMN25 | 0.873 | 0.952 | 0.916 | 174.4 | 0.8833 | 1.659 | 1.060 | 1.571 | 244.0 | 0.3521 |
| EMN200 - SPEECH0 | 2.810 | 0.814 | 3.451 | 93.9 | **0.0033** | 0.560 | 0.894 | 0.627 | 128.0 | 0.9650 |
| SPEECH0 - NOISE | -2.977 | 0.860 | -3.463 | 4477.2 | **0.0027** | 1.942 | 1.060 | 1.830 | 3641.0 | 0.2693 |
| EMN25 - NOISE | -1.040 | 0.992 | -1.049 | 4477 | 0.8833 | 0.844 | 1.200 | 0.702 | 3641.0 | 0.9650 |
| EMN200 - NOISE | -0.167 | 0.952 | -0.175 | 174.3 | 0.8833 | 2.503 | 1.080 | 2.311 | 269.0 | 0.1079 |

**Supplementary Table 7:** Results from linear mixed effects model estimates for intensity (dB). Note that sound levels were not meaningfully calibrated, so absolute dB numbers do not correspond to spoken sound pressure levels but relative values are meaningful. Separate models were fit for *3SYL* and for *SENT.* Separate models were fit for *3SYL* and for *SENT.* Reported *p*-values for individual fixed effects coefficients are not adjusted for multiple comparisons. Model formula: *INT ~ condition*delay + sex + age + trial + (1 + delay | subject*).

| | **3SYL** | | | | | **SENT** | | | | |
|---|---|---|---|---|---|---|---|---|---|---|
| *Fixed Effects* | *Estimates* | *CI* | *t* | *df* | *p* | *Estimates* | *CI* | *t* | *df* | *p* |
| intercept | 58.612 | 56.919 – 60.305 | 70.233 | 35.9 | **<0.001** | 56.034 | 54.581 – 57.488 | 78.206 | 35.7 | **<0.001** |
| condition [EMN25 - SPEECH0] | 0.957 | 0.653 – 1.261 | 6.171 | 4477.1 | **<0.001** | 1.452 | 1.145 – 1.759 | 9.275 | 3642.0 | **<0.001** |
| condition [NOISE - SPEECH0] | 1.247 | 0.942 – 1.551 | 8.035 | 4477.1 | **<0.001** | 1.575 | 1.260 – 1.890 | 9.804 | 3642.0 | **<0.001** |
| delay [SPEECH200 - SPEECH0] | 0.485 | 0.088 – 0.882 | 2.447 | 58.3 | **0.017** | 1.518 | 1.159 – 1.876 | 8.446 | 69.7 | **<0.001** |
| sex [MALE - FEMALE] | 4.460 | 1.206 – 7.714 | 2.782 | 35.0 | **0.009** | 3.048 | 0.229 – 5.867 | 2.195 | 35.0 | **0.035** |
| age (centered) | 0.190 | 0.059 – 0.322 | 2.950 | 35.0 | **0.006** | 0.133 | 0.019 – 0.246 | 2.369 | 35.0 | **0.024** |
| trial (centered) | 0.008 | 0.003 – 0.013 | 3.113 | 4498.8 | **0.002** | 0.005 | -0.002 – 0.011 | 1.480 | 3663.3 | 0.139 |
| condition x delay [EMN200 - EMN25] - [SPEECH200 - SPEECH0] | -0.450 | -0.842 – -0.057 | -2.247 | 4477.0 | **0.025** | -1.668 | -2.066 – -1.269 | -8.200 | 3642.0 | **<0.001** |
| | | | | | | | | | | |
| *Random Effects* | | | | | *Variance* | | | | | *Variance* |
| σ2 | | | | | 9.127 | | | | | 7.842 |
| τ00 SUBJECT | | | | | 19.399 | | | | | 14.105 |
| τ11 SUBJECT.delay200 | | | | | 0.885 | | | | | 0.573 |
| ρ01 SUBJECT | | | | | 0.245 | | | | | 0.138 |
| ICC | | | | | 0.696 | | | | | 0.653 |
| Marginal R2 | | | | | 0.215 | | | | | 0.157 |
| Conditional R2 | | | | | 0.761 | | | | | 0.708 |

**Supplementary Table 8:** Results of planned contrasts for intensity (dB). *P*-values are adjusted for multiple comparisons within family (the set of six contrasts, evaluated separately for *3SYL* and *SENT*) using the Bonferroni-Holm method.

| | ***3SYL*** | | | | | ***SENT*** | | | | |
|---|---|---|---|---|---|---|---|---|---|---|
| *Contrast* | *Estimate* | *SE* | *t* | *df* | *p* | *Estimate* | *SE* | *t* | *df* | *p* |
| SPEECH200 - SPEECH0 | 0.485 | 0.198 | 2.447 | 58.4 | 0.070 | 1.518 | 0.180 | 8.446 | 69.7 | **<0.0001** |
| EMN200 - EMN25 | 0.036 | 0.218 | 0.163 | 84.3 | 0.871 | -0.150 | 0.198 | -0.757 | 102.5 | 0.902 |
| EMN200 - SPEECH0 | 0.993 | 0.198 | 5.003 | 58.5 | **<0.0001** | 1.302 | 0.179 | 7.283 | 68.4 | **<0.0001** |
| SPEECH0 - NOISE | -1.247 | 0.155 | -8.035 | 4477.1 | **<0.0001** | -1.575 | 0.161 | -9.804 | 3642.0 | **<0.0001** |
| EMN25 - NOISE | -0.290 | 0.179 | -1.618 | 4477.0 | 0.317 | -0.123 | 0.182 | -0.675 | 3642.0 | 0.902 |
| EMN200 - NOISE | -0.254 | 0.218 | -1.168 | 84.3 | 0.493 | -0.273 | 0.201 | -1.354 | 109.5 | 0.536 |

**Supplementary Table 9:** Results from linear mixed effects model estimates for intensity modulation (within-trial intensity standard deviation in dB). Separate models were fit for *3SYL* and for *SENT.* Separate models were fit for *3SYL* and for *SENT.* Reported *p*-values for individual fixed effects coefficients are not adjusted for multiple comparisons. Model formula: *INTsd ~ condition*delay + sex + age + trial + (1 + delay | subject*).

| | 3SYL | | | | | SENT | | | | |
|---|---|---|---|---|---|---|---|---|---|---|
| *Fixed Effects* | *Estimates* | *CI* | *t* | *df* | *p* | *Estimates* | *CI* | *t* | *df* | *p* |
| Intercept | 9.778 | 9.062 – 10.493 | 27.700 | 36.4 | **<0.001** | 9.856 | 9.539 – 10.173 | 62.804 | 41.0 | **<0.001** |
| condition [EMN25 - SPEECH0] | 0.279 | 0.094 – 0.465 | 2.955 | 4477.1 | **0.003** | 0.150 | -0.026 – 0.325 | 1.670 | 3642.1 | 0.095 |
| condition [NOISE - SPEECH0] | 0.642 | 0.456 – 0.827 | 6.786 | 4477.1 | **<0.001** | 0.199 | 0.018 – 0.379 | 2.159 | 3642.0 | **0.031** |
| delay [SPEECH200 - SPEECH0] | 0.590 | 0.319 – 0.860 | 4.373 | 52.9 | **<0.001** | 0.401 | 0.207 – 0.595 | 4.111 | 76.0 | **<0.001** |
| sex [MALE - FEMALE] | -0.658 | -2.028 – 0.711 | -0.976 | 35.0 | 0.336 | 0.476 | -0.097 – 1.048 | 1.687 | 35.0 | 0.101 |
| age (centered) | 0.027 | -0.028 – 0.082 | 0.996 | 35.0 | 0.326 | 0.018 | -0.005 – 0.041 | 1.607 | 35.0 | 0.117 |
| trial (centered) | 0.004 | 0.001 – 0.007 | 2.729 | 4495.5 | **0.006** | 0.000 | -0.003 – 0.004 | 0.201 | 3666.0 | 0.841 |
| condition x delay [EMN200 - EMN25] - [SPEECH200 - SPEECH0] | -0.819 | -1.058 – -0.580 | -6.712 | 4477.0 | **<0.001** | -0.682 | -0.910 – -0.453 | -5.854 | 3642.0 | **<0.001** |
| | | | | | | | | | | |
| *Random Effects* | | | | | *Variance* | | | | | *Variance* |
| σ2 | | | | | 3.390 | | | | | 2.572 |
| τ00 SUBJECT | | | | | 3.424 | | | | | 0.619 |
| τ11 SUBJECT.delay200 | | | | | 0.465 | | | | | 0.147 |
| ρ01 SUBJECT | | | | | 0.262 | | | | | -0.315 |
| ICC | | | | | 0.540 | | | | | 0.189 |
| Marginal R2 | | | | | 0.033 | | | | | 0.037 |
| Conditional R2 | | | | | 0.556 | | | | | 0.219 |

**Supplementary Table 10:** Results of planned contrasts for intensity modulation (within-trial intensity standard deviation in dB). *P*-values are adjusted for multiple comparisons within family (the set of six contrasts, evaluated separately for *3SYL* and *SENT*) using the Bonferroni-Holm method.

| | *3SYL* | | | | | *SENT* | | | | |
|---|---|---|---|---|---|---|---|---|---|---|
| *Contrast* | *Estimate* | *SE* | *t* | *df* | *p* | *Estimate* | *SE* | *t* | *df* | *p* |
| SPEECH200 - SPEECH0 | 0.590 | 0.135 | 4.373 | 52.9 | **0.0003** | 0.401 | 0.098 | 4.111 | 76.0 | **0.0006** |
| EMN200 - EMN25 | -0.229 | 0.145 | -1.574 | 71.5 | 0.2398 | -0.281 | 0.109 | -2.588 | 116.2 | **0.0436** |
| EMN200 - SPEECH0 | 0.050 | 0.135 | 0.373 | 53.0 | 0.7106 | -0.131 | 0.097 | -1.352 | 74.4 | 0.3609 |
| SPEECH0 - NOISE | -0.642 | 0.095 | -6.786 | 4477.1 | **<0.0001** | -0.199 | 0.092 | -2.159 | 3642.0 | 0.0927 |
| EMN25 - NOISE | -0.362 | 0.109 | -3.322 | 4477.0 | **0.0027** | -0.049 | 0.104 | -0.470 | 3642.0 | 0.6387 |
| EMN200 - NOISE | -0.591 | 0.145 | -4.065 | 71.5 | **0.0005** | -0.330 | 0.111 | -2.983 | 124.8 | **0.0171** |
